# Hypoxia tolerance determine differential gelsenicine-induced neurotoxicity between pig and mouse

**DOI:** 10.1186/s12916-025-03984-5

**Published:** 2025-03-12

**Authors:** Chong-Yin Huang, Meng-Ting Zuo, Xue-Jia Qi, Meng-Die Gong, Wen-Bo Xu, Si-Yu Meng, Jiang-Yu Long, Pi-Shun Li, Zhi-Liang Sun, Xiao-Feng Zheng, Zhao-Ying Liu

**Affiliations:** 1 College of Veterinary Medicine, Hunan Agricultural University, Changsha, 410128 China; 2 Hunan Engineering Technology Research Center of Veterinary Drugs, Hunan Agricultural University, Changsha, 410128 China

**Keywords:** *Gelsemium*, Gelsenicine, Glycine, Excitotoxicity, NMDA receptors

## Abstract

**Background:**

*Gelsemium elegans* (*G. elegans*) is widely recognized as one of the most toxic plants globally, particularly harmful to humans. Some reports indicate that it is non-toxic to pigs and even has a growth-promoting effect; however, the underlying reasons for this paradox remain unclear.

**Methods:**

Gelsenicine is the main toxic component of *G. elegans*. This study characterized gelsenicine-induced toxicity using electrophysiological recordings, molecular dynamic simulations, c-Fos immunostaining, and multi-omics technologies. Additionally, we conducted a comprehensive analysis comparing the toxic effects of gelsenicine across various animal species through examinations of tissue distribution, blood gas analysis, metabonomics, and behavioral tests.

**Results:**

We demonstrated that gelsenicine-induced hypoxia leads to respiratory depression in mice by enhancing the effect of gamma-aminobutyric acid (GABA) on GABA receptors (GABARs). Glycine significantly ameliorated hypoxia and improved the survival of gelsenicine-poisoned mice. Under gelsenicine-induced hypoxic conditions, N-methyl-D-aspartate (NMDA) receptor function and mitochondrial energy metabolism processes were perturbed, resulting in neuronal excitotoxicity. Finally, we confirmed that pigs could tolerate hypoxia and were resistant to gelsenicine toxicity due to high concentrations of circulating glycine and low levels of NMDA receptors (NMDARs) in the hippocampus.

**Conclusions:**

These findings suggest that hypoxic protection should be considered as a potential therapeutic strategy for gelsenicine poisoning. Our study contributes to preventing potential risks posed by *G. elegans* poisoning to human and animal health.

**Graphical Abstract:**

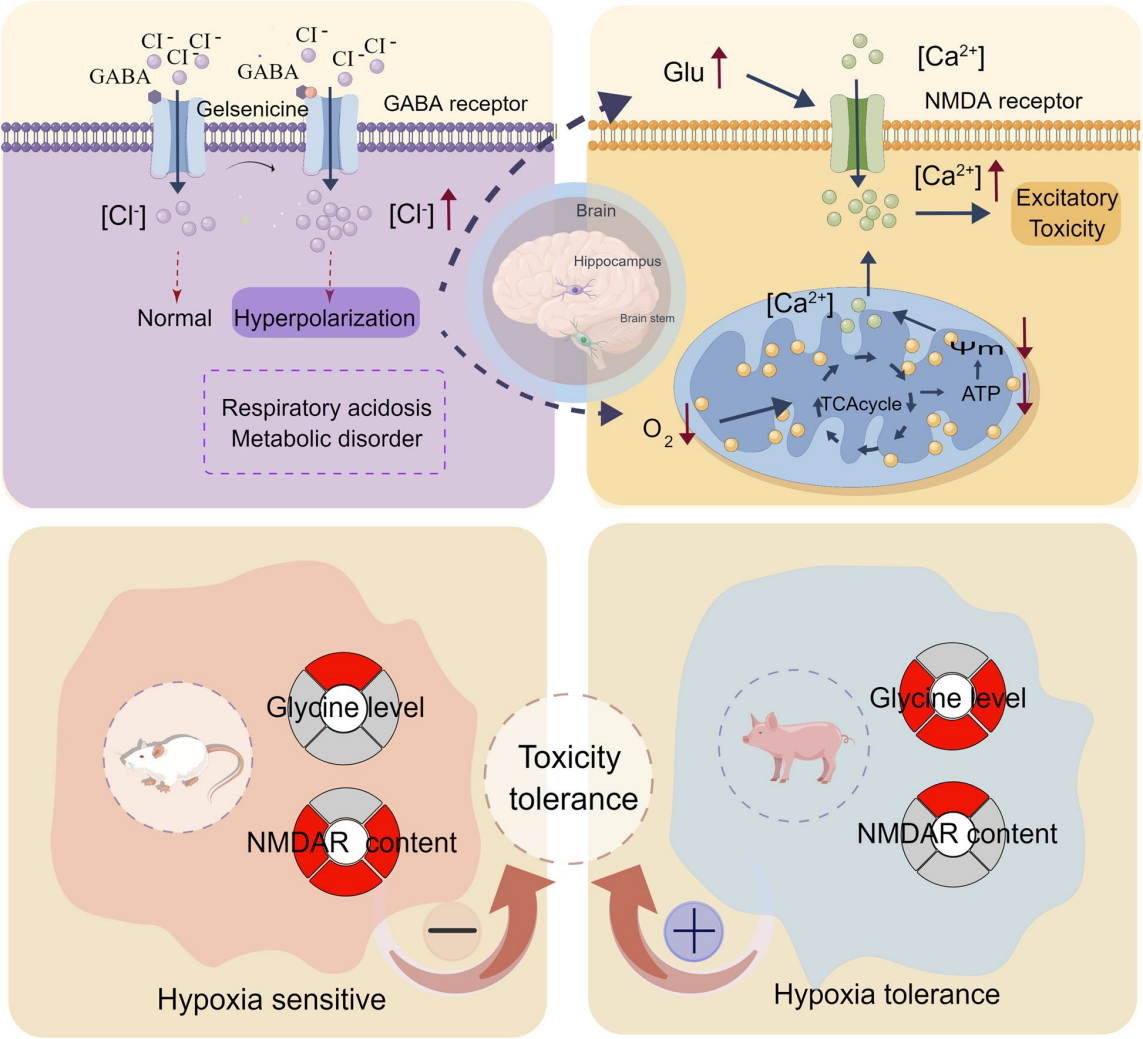

**Supplementary Information:**

The online version contains supplementary material available at 10.1186/s12916-025-03984-5.

## Background

*Gelsemium*, a whole grass in the family Gelsemiaceae [1], is a poisonous plant worldwide that comprises three species [2]. *Gelsemium sempervirens* and *Gelsemium rankinii*, native to North America, are well-known for their use as homeopathic remedies [3]. The Asian variant, *Gelsemium elegans* (*G. elegans*), has been a traditional Chinese herbal medicine for many years to treat neuropathic pain, rheumatoid pain, inflammation, skin ulcers, and cancer [4–8]. However, frequent cases of acute *G. elegans* poisoning are a significant concern, particularly in rural China [9, 10]. When it is ingested improperly or in excessive amounts, *G. elegans* causing severe neurotoxicity (including seizures, dizziness, and coma), respiratory distress, heart failure, and even death [11, 12]. To date, no effective targeted drugs have been developed to counteract poisoning from *G. elegans*. In such circumstances, *G. elegans*-induced toxicity has garnered considerable attention.

The main components of *G. elegans* are indole alkaloids, among which gelsenicine is the primary toxic constituent [13]. Notably, pigs exhibit a significant resistance to the toxicity induced by *G. elegans* and its alkaloids. A large amount of evidence has confirmed that *G. elegans* is even widely used as a feed additive to improve the growth performance of pigs in Chinese folk [14–17]. However, people accidentally ingest small amounts of *G. elegans* leaves, resulting in poisoning [9, 18]. Mice exhibit a high sensitivity to gelsenicine, with the 50% lethal dose (LD_50_) of 0.185 mg/kg [13]. The reasons for this difference in results remain uninvestigated.

Previous studies have shown that the balance between excitation and inhibition is important for maintaining neural stability [19]. Prolonged activation of GABAR and excessive excitement of NMDAR both may produce neurotoxic effects by disturbing the delicate balance between excitation and inhibition [20]. Recent studies have demonstrated a strong relationship between gelsenicine and the GABAR [21, 22]. Our previous electrophysiological data revealed that gelsenicine could regulate GABA_A_Rs and significantly prolong the opening time of *chloride* channels [23, 24]. Except for GABAR, NMDAR-dependent excitotoxicity is a critical pathway in gelsenicine-induced neurotoxicity [13]. However, the relationship between there is no direct evidence linking the action of gelsenicine on these membrane receptors to its neurotoxicity effects. The mechanism of gelsenicine-induced neurotoxicity still needs to be explored.

To address these questions, we conducted experiments using various approaches, including electrophysiological recordings, behavioral tests, molecular dynamic simulations, c-Fos immunostaining, and multi-omics technologies. Here, we not only elucidated the mechanism by which gelsenicine acts on membrane receptors, thereby uncovering its neurotoxic effects, but also explored possible causes for the differential gelsenicine-induced toxicity of species. Moreover, we identified a previously unknown effective drug for gelsenicine poisoning. The newly understood mechanism of gelsenicine-induced toxicity provides pivotal information for the development of therapeutic strategies to overcome *G. elegans* poisoning.

## Methods

### Pig and mouse models

Ethical committee number for the study: 2023–022 and 2020–43. All animal studies were performed following the national legislation and were approved by the Institutional Animal Care and Use Committee at the Center for Laboratory Animals, Hunan Agricultural University.

SPF grade ICR male mice (neonatal and adult); SD male rats weighing 200 ± 20 g, were purchased by Slake Jingda Laboratory Animal Technology company Hunan province (SCXK2021–0002). Neonatal mice were 1-week-old; Adult mice were 6–8-week-old. All animals were housed in a constant temperature environment (25 ± 1ºC) with lighting from fluorescent lamps alternating every 12 h. Animals had free access to food and water.

Male ternary hybrid piglets, weighing 5–10 kg, were purchased from the Xin Guangan Xiangda Co.Ltd. They were provided with sufficient basic feed and clean water, and were acclimatized for one week before the start of the experiment.

### Cell culture

Cell Culture: In this study, HEK-293 cell lines stably expressing GluN1/GluN2A or GluN1/GluN2B receptors were used. The GluN1, splice variant NM_001114183.2. The HEK-293 cell lines stably expressing GluN1/GluN2A or GluN1/GluN2B receptors were cultured in Dulbeccos modified Eagles medium (DMEM) containing 10% fetal bovine serum, 10 µg/mL Blasticidin, 100 µg/mL Zeocin, and 200 µg/mL Hygromycin B, at 37 °C with a CO_2_ concentration of 5%.

Cell passage: The old culture medium was removed and washed once with PBS. Then, 1 mL of 0.25% Trypsin–EDTA solution was added and incubated at 37 °C for about 0.5 min. When the cells detached from the dish, add about 5 mL of preheated complete culture medium at 37 °C. Gently pipette the cell suspension to disperse the cell aggregates. Transfer the cell suspension to a sterile centrifuge tube and collect the cells by centrifugation. For amplification or maintenance of culture, the cells were seeded into 6 cm cell culture dishes at a density of 2.5 × 105 cells per dish (final volume: 5 mL). To maintain the electrophysiological activity of the cells, the cell density should not exceed 80%.

Prior to electrophysiological recordings, cells were dissociated using 0.25% Trypsin–EDTA, and 8 × 10^3^ cells were seeded onto a coverslip in a 24-well plate (final volume: 500 µL). The cells were induced with doxycycline and treated with 1 mM D(-)−2-Amino-5-phosphonovaleric acid (DAP-5). After 18 h, the experimental measurements were performed.

### Study on the toxicity of gelsenicine

To investigate the potential toxicity of gelsenicine, various doses of gelsenicine (ranging from 0.12 to 0.24 mg/kg) were administered intraperitoneally (i.p.) to mice (neonatal and adult). In addition, to evaluate the variation in gelsenicine toxicity among different animal species, mice and rats were orally administered gelsenicine at doses ranging from 1 to 2 mg/kg, whereas pigs were administered gelsenicine orally at doses ranging from 2 to 10 mg/kg.

After drug administration, the main toxic symptoms of the animals were observed immediately, and all deaths, latency period, severity, duration, and time of death of any toxic reactions were recorded. The incidence rate (number of animals with toxic reactions/total sample size) and mortality rate (number of deaths/total sample size) were calculated.

### Blood analysis

For blood routine examination, 50 μL fresh blood was collected from each mouse and mixed with EDTA immediately. For blood gas analysis, blood samples from an artery were collected. The physiological parameters, including red blood cell, hemoglobin, mean corpuscular hemoglobin concentration, arterial pH, arterial partial pressure of carbon dioxide (PaCO_2_), and arterial partial pressure of oxygen (PaO_2_) were monitored in mice and pigs before and after gelsenicine administration using DF-50 series all automatic hematology analyzers (Dymind Biotechnology Co., Ltd, Shenzhen, China) and Radiometer blood gas analyzer (Shenzhen Libang Precision Instrument Co., Ltd., Shenzhen, China).

### Effect of inhibition of P-gp on permeability and toxicity of gelsenicine blood–brain barrier

Fourteen male ICR mice were randomly divided into the gelsenicine group and gelsenicine + P-glycoprotein (P-gp) inhibitors tariquidar (A8208, Apexbio, Shanghai, China) combined group. They were given oral gavage of blank solvent and tariquidar (20 mg/kg) separately, followed by gelsenicine (i.p. 0.12 mg/kg) after 30 min. The mice were observed for their status and behavior during the experiment and 1 h after the experiment, and all deaths were recorded.

### Acute toxicity of drug against gelsenicine in mice

Male ICR mice (7 mice for each set of experiments) were randomly assigned to the groups. Different neurotransmitter compounds, including glycine, sarcosine, GABA, MgSO_4_, and NMDA, were administered via i.p. injections. After 20 min, the groups were injected with a solution of gelsenicine (0.24 mg/kg) with a volume of 0.1 mL/10 g. Reduced mouse activity, weakness, and prostration were signs of poisoning. The onset, duration, and time of death from poisoning reactions within 1 h were recorded for the groups.

### Sample collection

Blood samples were collected from each experimental animal in heparinized tubes before euthanasia. After euthanasia, the chest, abdomen, and skull were quickly opened to collect the following tissues: heart, lungs, liver, spleen, kidneys, pancreas, spinal cord, intestines, muscles, testicles, cecum contents, and different brain regions, including the hippocampus, striatum, cortex, cerebellum, brainstem, and hypothalamus. The euthanasia times for each experimental group were as follows: solvent control and non-intoxicated groups were euthanized 20 min after gavage. The intoxicated group was euthanized at 20 min, 40 min, and 1, 2, and 4 h after administration. The death group was euthanized when the animals exhibited severe toxic reactions and were close to death.

After collection, blood was transferred to a heparin sodium anticoagulant tube and placed on ice. Then, the plasma was quickly separated by centrifugation, transferred into cryotubes, and stored at –80 °C. The remaining tissue and brain samples were immediately snap-frozen in liquid nitrogen and stored at –80 °C.

### Detection of distribution of tissue

Gelsenicine levels of plasma and tissue samples were determined according to our previous study [14, 25–27]. In brief, central and peripheral samples were processed by homogenization and centrifugation. Next, a series of standard solutions of gelsenicine were prepared with methanol, and the concentrations were 100 μg/mL, 50 μg/mL, 20 μg/mL, 10 μg/ml, 5 μg/mL, 2 μg/mL, 1 μg/mL, 500 ng/mL, 200 ng/mL and 0 ng/mL respectively. Finally, all samplers were further analyzed using two-dimensional liquid chromatography with Ultraviolet detection (2D-LC-UV).

### Metabolomics test

Metabolome analysis was performed by Shanghai Zhongke New Life Biotechnology Co., Ltd, as described previously [28]. Samples were collected from four groups, including pig control group, pig gelsenicine-treated group (6 mg/kg), rat control group and rat gelsenicine-treated group (1.2 mg/kg), with 8 animals in each group. The sampling time was determined to be 20 min following administration. 100 uL fozen plasma metabolites were extracted in accordance with the provided protocol [29]. The hippocampus samples of the blank group and gelsenicine poisoning group (20 min) were thawed at 4 ℃, and 100 mg was taken and ground with liquid nitrogen. The mixture was vortexed 2–3 times for 1–2 min each time, followed by ice bath ultrasonication for 60 min and incubation at –20 ℃ for 1 h. The mixture was then centrifuged at 14,000 g, 4 ℃ for 15 min to precipitate proteins. The supernatant was freeze-dried and reconstituted with 100 μL of acetonitrile–water solution (1:1, v/v). A 2 μL aliquot of the supernatant was filtered through a 0.22 μm cellulose membrane and injected into an autosampler for liquid chromatography-tandem mass spectrometry (LC–MS/MS) analysis.

Then, the original MS data was converted to MzXML files and imported into the free available XCMS software (https://sciex.com/products/software/xcms-plus-software) using the ProteoWizard MSConvert tool (http://proteowizard.Sourceforge.net/). Chromatographic peaks were filtered using the following parameters: peak deviation m/z = 25 ppm, peak width = c (10, 60), prefilter = c (10, 100). Peak grouping was performed with parameters bw = 5, mzwid = 0.025, minfrac = 0.5. Collection of Algorithms of MEtabolite pRofile Annotation (CAMERA) was used for isotope and adduct annotation. In the extracted ion features, only variables with at least one group of non-zero measurement values and present in more than 50% of the samples were retained. Compound identification of metabolites was performed by comparing the m/z values (< 25 ppm) and MS/MS spectra with the available internal standard compound database. Data were normalized to total peak intensity and exported to SIMCA-P software (http://www.umetrics.com/simca) for multivariate data analysis to screen for differential metabolites.

### TTC staining

After completing the above tests, the brains of the mice in the groups were removed by decapitation, and heart tissues were collected and washed with physiological saline. The tissues were then placed in a –80 ℃ freezer for 5 min to freeze them. The brain tissue was sliced coronally at a thickness of 2 mm from the anterior to posterior direction, and the heart was sliced parallel to the coronary sulcus to obtain the ventricular part. The sliced tissues were immediately placed in a 2% 2,3,5-triphenyltetrazolium chloride (TTC) staining solution, incubated at 37 ℃ in the dark for 20 min, and then fixed in 4% paraformaldehyde at 4 ℃ for 12 h. In viable tissue, TTC is metabolized by mitochondria to a red compound, triphenylformazan, the red areas indicate the living tissue, while the ischemic area remains colorless. The stained sections were recorded using a digital camera and quantified for each slice's optical density and total area using Image Pro Plus 6.0 software (Media Cybernetics, Bethesda, MD, USA). The average optical density (total IOD/total area) was used as the statistical parameter.

### Immunofluorescence detection of c-Fos protein in hippocampus

The mice were euthanized and the brains were postfixed overnight in 4% paraformaldehyde at 4 °C. The brains were then immersed in a 30% sucrose solution for 48 h. Brains were then frozen and sliced in 5 μm coronal sections. Every hippocampus was used for c-fos immunoreactivity (FOS-ir) staining, using the avidin–biotin-peroxidase technique. Antigen retrieval was performed with ethylenediaminetetraacetic acid (*pH9*) at high pressure and temperature for 90 s with subsequent blocking in 3% H_2_O_2_ at room temperature for 30 min_._ Sections were washed and incubated in 10% normal goat serum for 30 min at room temperature. Brain slices were incubated in rabbit anti-c-Fos (1:1000, Abcam, #ab222699) overnight at 4 °C and then with secondary antibody goat anti-rabbit-Horseradish Peroxidase (HRP) (1:2000, Abcam, #ab205718) for 45 min at 37 °C. A nickel/diaminobenzidine (DAB) reaction was used to visualize c-Fos immunoreactivity [30]. The nucleus of the hematoxylin stained is blue, and the positive expression of DAB is brownish yellow. All images were viewed and captured with light microscopy (Olympus, #CX31). *NDP.vew 2* software was used to process the images. C-Fos-positive cells in the cellular layers of the hippocampus were manually counted by a trained observer blind to the treatment.

### Patch-clamp detection of stably overexpressed GluN1/GluN2A and GluN1/GluN2B currents

The experiments were conducted using an EPC-10 patch-clamp amplifier (HEKA, Germany) to perform whole-cell recording under conventional voltage-clamp mode. Using a HEK-293 cell line with stable expression of GluN1/GluN2A or GluN1/GluN2B receptors, the cell membrane voltage clamp was applied at –70 mV when a whole-cell seal was formed. The number of cells was 2 as the number of replicates of this experiment. The composition of the extracellular fluid was as follows: 140 mM NaCl, 4 mM KCl, 2 mM CaCl_2_•2H_2_O, 10 mM HEPES, 5 mM D-Glucose, NaOH was used to adjust the pH to 7.4. The composition of the intracellular fluid was as follows: 10 mM NaCl, 110 mM CsMes, 2 mM MgCl_2_•6H_2_O, 10 mM HEPES, 10 mM EGTA, 2 mM Na2-ATP, 0.2 mM Na2-GTP, CsOH was used to adjust the pH to 7.2.

The inhibitory effect of the galsenicine on NMDAR currents was performed: In Gap-free mode, Glycine 10 µM + L-Glutamate 10 µM was sprayed on the cell surface successively and recorded for about 20 s after the current stabilized. Then the mixed working solution of Glycine 10 µM + L-Glutamate 10 µM + gelsenicine was given and recorded for about 20 s to observe the inhibitory effect of the drug on GluN1/GluN2A or GluN1/GluN2B currents. Finally, the mixed working solution of Glycine 10 µM + L-Glutamate 10 µM + D-AP5 100 µM was given to observe the inhibitory effect of current.

The excitatory effect of the galsenicine on NMDAR currents was recorded: In Gap-free mode, the cell surface was rapidly sprayed with Glycine 10 µM + the working liquid mixture of the tested compound, and the cell surface was recorded for about 20 s. After the current was stable, Glycine 10 µM + L-Glutamate 10 µM was given again. The effect of the drug on GluN1/GluN2B current was observed. Finally, the mixed working solution of Glycine 10 µM + L-Glutamate 10 µM + D-AP5 100 µM was given to observe the inhibitory effect of current.

The test data was collected by EPC 10 amplifier and stored in PatchMaster software (HEKA, Lambrecht, Germany, https://www.heka.com/downloads/downloads_main.html). All electrophysiological tests were performed at room temperature. Data acquisition and analysis were performed using IGOR Pro software. The whole-cell recording data were analyzed and plotted using PatchMaster and IGOR Pro software. NMDAR peak currents were normalized to compare the differences in normalized currents between treatment groups. The method for calculating the normalized NMDAR peak current was: Normalized NMDA peak current = I / I_NMDA_, where I_NMDA_ represents the peak current of the NMDAR before drug application, and I represents the peak current of the NMDAR after treatment with the target drug or antagonist.

### Molecular docking of gelsenicine and GABAR

Retrieve the three-dimensional structure of protein gelsenicine from the PubChem database (https://PubChem.ncbi.nlm.nih.gov) and perform energy minimization on it using Chem3D software (Cambridge Soft, Cambridge, MA, USA). The 3D structure of protein human GABA_A_R α1β2γ2 subunit (PDB code 6D6T) was downloaded from the PDB database (http://www.rcsb.org). The protein was placed in bilayers composed of 512 POPC lipid (1-palmitoyl-2-oleoyl phosphatidylcholine) molecules using the CHARMM-GUI online tool (https://charmm-gui.org/). The lipid molecules are located in the xy plane and the protein molecules are perpendicular to the lipid bilayer plane. The receptor protein was then dehydrated and modified with PyMOL 3 software (www.schrodinger.com/pymol). Autodock Tools 1.5.6 software was used to calculate the hydrogen bond and charge of the protein. The parameters of the receptor protein's docking site included the active pocket site for the binding of small-molecule ligands. Finally, the GABAR protein with gelsenicine was docked by AutoDock Vina 1.5.6 [31].

### Molecular docking studies on species difference in the toxicity of gelsenicine

Retrieve and download the amino acid sequences of GABA_A_R from pig, rat, and mouse sources from the NCBI database (https://www.ncbi.nlm.nih.gov/). We utilized the SWISS-MODEL online server (http://swissmodel.expasy.org/) to construct the three-dimensional structures of GABA_A_R from pig, rat, and mouse, using the human GABA_A_R complexed with flumazenil (PDB code 6D6T) as a template. This template showed high sequence identities of 99.89% for pig, 100% for rat, and 100% for mouse, which facilitated the modeling process. After rigorous model evaluation, we proceeded to perform docking studies using AutoDock Vina, exploring the interactions between GABA_A_Rs from these species and gelsenicine.

### Transmission electron microscopic experiment of hippocampal region

The change in mitochondrial ultrastructure were confirmed by transmission electron microscopy (TEM). The fresh hippocampus tissues were fixed with TEM fixation solution and post-fixed with 1.0% (w/v) osmium tetroxide in the same buffer, followed by dehydration in a graded series of ethanol, propylene oxide treatment, and then embedded in epoxy resin, and sectioned. The ultrathin sections were stained with uranyl acetate and leas citrate, dried at room temperature overnight, and observed under TEM to collect images for analysis.

### Western blot

Hippocampus samples were collected for subsequent analyses. Total protein was extracted from tissues using RIPA lysis buffer (Coolaber, Beijing, China) supplemented with protease inhibitor and phosphatase inhibitor. The concentration of the extracted proteins was determined using the bicinchoninic acid method (Coolaber, Beijing, China).

Protein samples are first separated by SDS-PAGE gel electrophoresis. Subsequently, the separated proteins are transferred onto a PVDF membrane. The membrane is then blocked by blocking buffer (EpiZyme, Shanghai, China) and and incubated with the primary antibodies (1:1000) overnight at 4 °C. Following that, the Goat anti-rabbit secondary antibody (1:5000, Biodragon, Suzhou, China) is used. Finally, Enhanced Chemiluminescence (EpiZyme, Shanghai, China) are employed to visualize and quantify protein bands.

### Determination of energy metabolism index

Hippocampus, brainstem, and blood samples were collected for subsequent analyses. The lactate content (Solarbio, Beijing, China), ATP content (Solarbio, Beijing, China), Na^+^-K^+^-ATPase activity (Solarbio, Beijing, China) and Ca^2+^ (Ruixin Blotech, Fujian, China) were analyzed using commercial kits according to the instructions.

The ATP content can reflect the status of energy metabolism. For ATP content assay, tissue from fresh mouse hippocampus tissue blocks was lysed with ATP lysis buffer and subjected to thorough grinding. This tissue was then centrifuged at 8,000 × *g* at 4 ℃ for 10 min. The sample supernatant was transferred to a new tube, 500 μL of chloroform was added and mixed thoroughly by vortexing, and then centrifuged at 10,000 × *g* at 4 ℃ for 3 min. The sample supernatant was transferred to a new tube for ATP detection. The ATP content was measured with HET-013 Multifunctional Microplate Reader (BIOTEK, USA).

Na^+^-K^+^-ATPase that maintain the gradient of Na^+^ and K^+^ across the cell membrane, their activity is one of the indicators for evaluating the function of the neuronal plasma membrane [32]. For Na^+^-K^+^-ATPase activity assay, ATPase activity was determined by the amount of phosphorus generated by ATPase and substrate. Tissue from fresh mouse hippocampus tissue blocks was lysed with lysis buffer and subjected to thorough grinding. This tissue was then centrifuged at 8,000 × *g* at 4 ℃ for 10 min. The sample supernatant was transferred to a new tube for Na^+^-K^+^-ATPase activity detection. The Na^+^-K^+^-ATPase activity was measured with HET-013 Multifunctional Microplate Reader.

Methyl thymol blue forms a chromogenic complex with Ca^2+^, was used analyze calcium salts in cells and tissues. Fresh mouse hippocampal tissue was mixed with deionized water and subjected to thorough grinding. This tissue was then centrifuged at 8,000 × *g* at 4 ℃ for 10 min. The sample supernatant was transferred to a new tube for Ca^2+^ detection. The Ca^2+^ content was measured with HET-013 Multifunctional Microplate Reader and normalized to the protein content.

### Determination of mitochondrial membrane potential

Hippocampus mitochondria were isolated using the mitochondria isolation kit (Solarbio, Beijing, China). The mitochondrial membrane potential was measured by mitochondrial membrane potential assay kit (Solarbio, Beijing, China). The principle was based on the mitochondrial membrane potential's effect on JC-1 fluorescence. When the mitochondrial membrane potential was high, JC-1 aggregates within the mitochondrial matrix to form polymers, resulting in red fluorescence. Conversely, when the mitochondrial membrane potential was low, JC-1 remains in its monomeric form and does not aggregate within the matrix, emitting green fluorescence. This change from red to green fluorescence indicates the loss of mitochondrial membrane potential, as detected by the fluorescent cationic dye JC-1 [33]. Briefly, the mitochondria were loaded with 1 × JC-1 dye at 37 °C for 30 min, and then analyzed, after washing, by a fluorescent enzyme analyzer. The proportion of mitochondrial depolarization was measured based on the relative ratio of red and green fluorescence.

### Statistical analysis

All data were presented as mean ± standard deviation (SD). Statistical analysis was made using GraphPad Prism 8 software (http://www.graphpad.com/). Student t test was employed to analyze the statistical significance between 2 groups. For more than two groups, one-way ANOVA was used. *P* < 0.05 was considered to be statistically significant.

## Results

### Central respiratory depression produced by gelsenicine

**Fig. 1:**
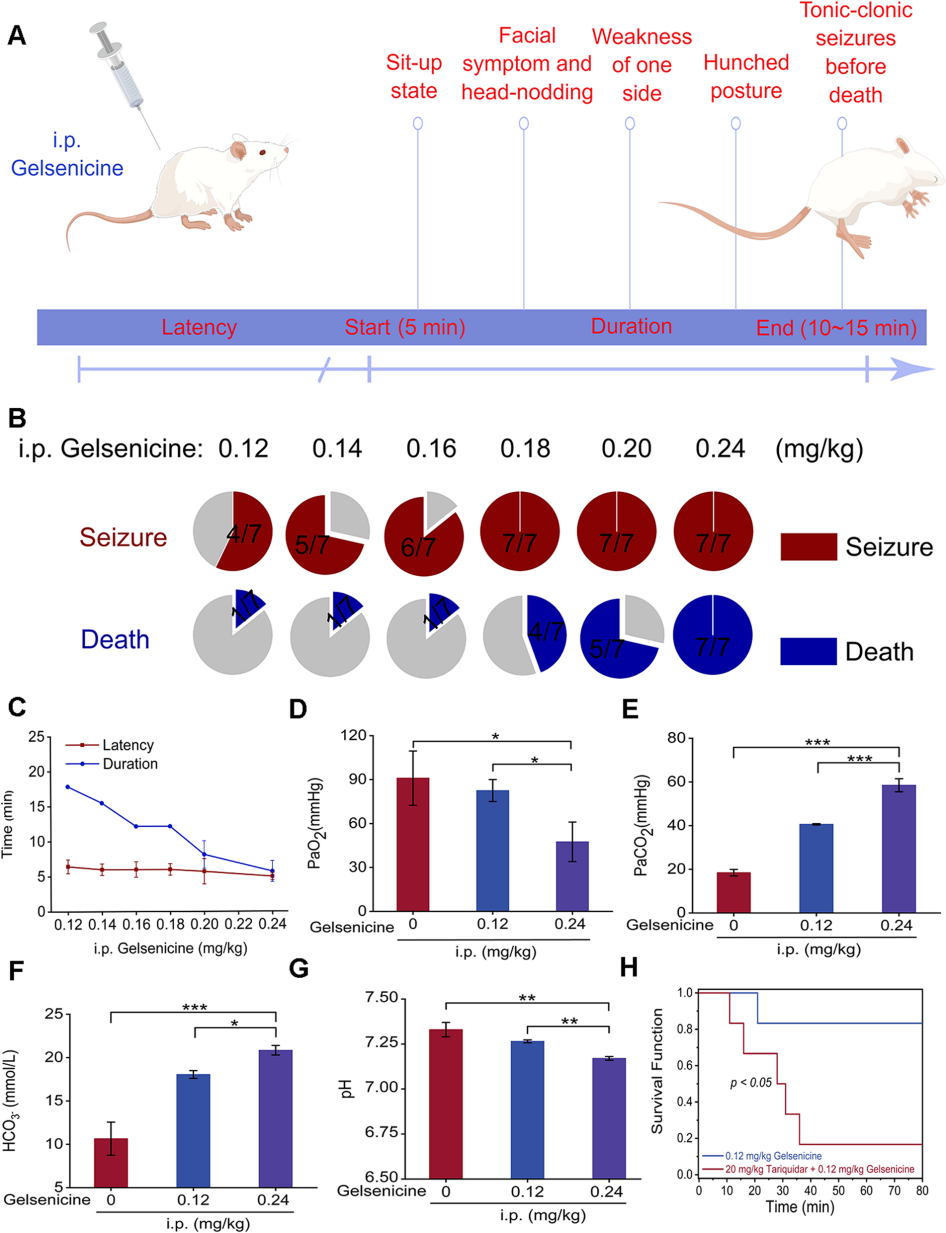
Effects of gelsenicine toxicity. **A** Schematic diagram of gelsenicine intoxication in mice by i.p. injection. The figure was generated by Figdraw (www.figdraw.com). **B** Pie charts depicting toxicity and mortality caused by i.p. injection of gelsenicine at various doses (0.12–0.24 mg/kg). *n* = 7 mice/group. **C** Dose responses of i.p. gelsenicine on reaction time (latency) and death time 1 (**D**–**G**) Alterations in the blood-gas parameters derived from arterial blood samples. *n* = 3 mice/group. **H** Survival curve of P-gp inhibition model (by using of 20 mg/kg of Tanquidar) in low-dose (0.12 mg/kg) gelsenicine-infected mice. *n* = 7 mice/group. Data are presented as mean ± SD. *P < 0.05, **P < 0.01, *** P < 0.001 based on one-way ANOVA; Pa, partial pressure

Continuous monitoring observational studies were performed to characterize the development of gelsenicine toxicity in mice at different doses (Fig. 1A, B). A relatively high dose (> 0.16 mg/kg) of gelsenicine administered through i.p. injection could induce obvious poisoning symptoms in most mice, including decreased ambulatory movements, limb weakness and other inhibition symptoms within 5 min of poisoning, followed by a gradual progression to an excitatory state, accelerated respiratory rate, obvious head tremor, rhythmic spasm in back and abdomen, and a comprehensive compulsory spasm attack at the end, and the mice exhibited as convulsion and jump. This is consistent with previous reports on rats [1, 34]. The incubation period and poisoning time for gelsenicine toxicity were recorded. The findings (Fig. 1C) indicated that acute poisoning caused the death of the mice, and the mortality rate accelerated with an increase in poisoning dose, implying a dose–response relationship for gelsenicine poisoning. These results suggest that gelsenicine causes acute toxicity in mice, with neurological and respiratory symptoms as the primary manifestations of gelsenicine toxicity.

To understand the effect of gelsenicine on the respiratory system, blood gas analysis was performed on left ventricular arterial blood. PaO_2_ decreased and then returned to normal in mice administered with a low dose of gelsenicine (0.12 mg/kg), indicating transient respiratory toxicity. Following gelsenicine administration (i.p. 0.24 mg/kg) for 5 min, an aggravation in the respiratory function of the mice was observed. Specifically, the arterial blood sample appeared dark red, and the PaO_2_ decreased rapidly from approximately 91 to 47.5 mmHg (*P* < 0.05) (Fig. 1D). Concurrent with this decrease in PaO_2,_ PaCO_2_ increased significantly from 18.45 to 58.5 mmHg (*P* < 0.01) (Fig. 1E). The PaCO_2_ was approximately five times higher than the normal level at 10 min (Additional file 1: Table S1). Our results suggest that gelsenicine significantly impaired respiratory function and induced acute death in mice. In addition, the observed increase in HCO_3_^−^ (Fig. 1F) and the corresponding decrease in pH (Fig. 1G) suggest an acid–base balance disorder in the animals, such as acute respiratory acidosis. Anion gap (AG) is an important indicator for distinguishing between respiratory and metabolic acidosis [35]. Our findings revealed that AG increased to 22.33 mmol/L in the later stage (*P* < 0.01) (Additional file 1: Table S1), suggesting that the metabolic disorder was caused by respiratory depression in mice, followed by metabolic acidosis. Although poisons can cause acute respiratory dysfunction by affecting the ability of the blood to absorb or transport oxygen, it is unlikely that gelsenicine toxicity is mediated through blood supply factors. This is supported by evidence that gelsenicine does not affect hematological parameters (Additional file 1: Table S2).

Previous studies have demonstrated the presence of gelsenicine in rat brains, indicating its potential to cross the blood–brain barrier (BBB) [36]. However, in this process, the majority of drugs rely on transporters within the BBB, with P-gp playing an especially significant role. P-gp, an efflux transporter located on the BBB, functions to block or limit the entry of potentially harmful substances into the brain [37]. To further explore the main organ of gelsenicine toxicity, we selected tariquidar to specifically inhibit P-gp transporters in the BBB and increase drug exposure to the brain. The mortality rate was evaluated after administration of low-dose gelsenicine alone and in combination with P-gp inhibitors. We identified that low-dose gelsenicine (i.p. 0.12 mg/kg) did not result in significant mortality in mice (Fig. 1H). However, pretreatment with P-gp inhibitors exacerbates gelsenicine-induced toxicity, leading to a significant increase in mortality in mice (*P* < 0.05). Specifically, the mortality rate was 3.92 times higher than that in the low-dose gelsenicine group. Our findings suggest that P-gp plays a crucial role in regulating the BBB permeability and the central effect of gelsenicine. Inhibition of the P-gp efflux transporters in mice led to a reduction in the exclusion of gelsenicine from the brain and significantly exacerbated toxicity. Based on these observations, we speculated that the brain is likely to be the primary site of gelsenicine toxicity.

### The high tolerance of pig brain to the toxic effects of gelsenicine is likely due to its ability to tolerate hypoxic conditions

**Fig. 2:**
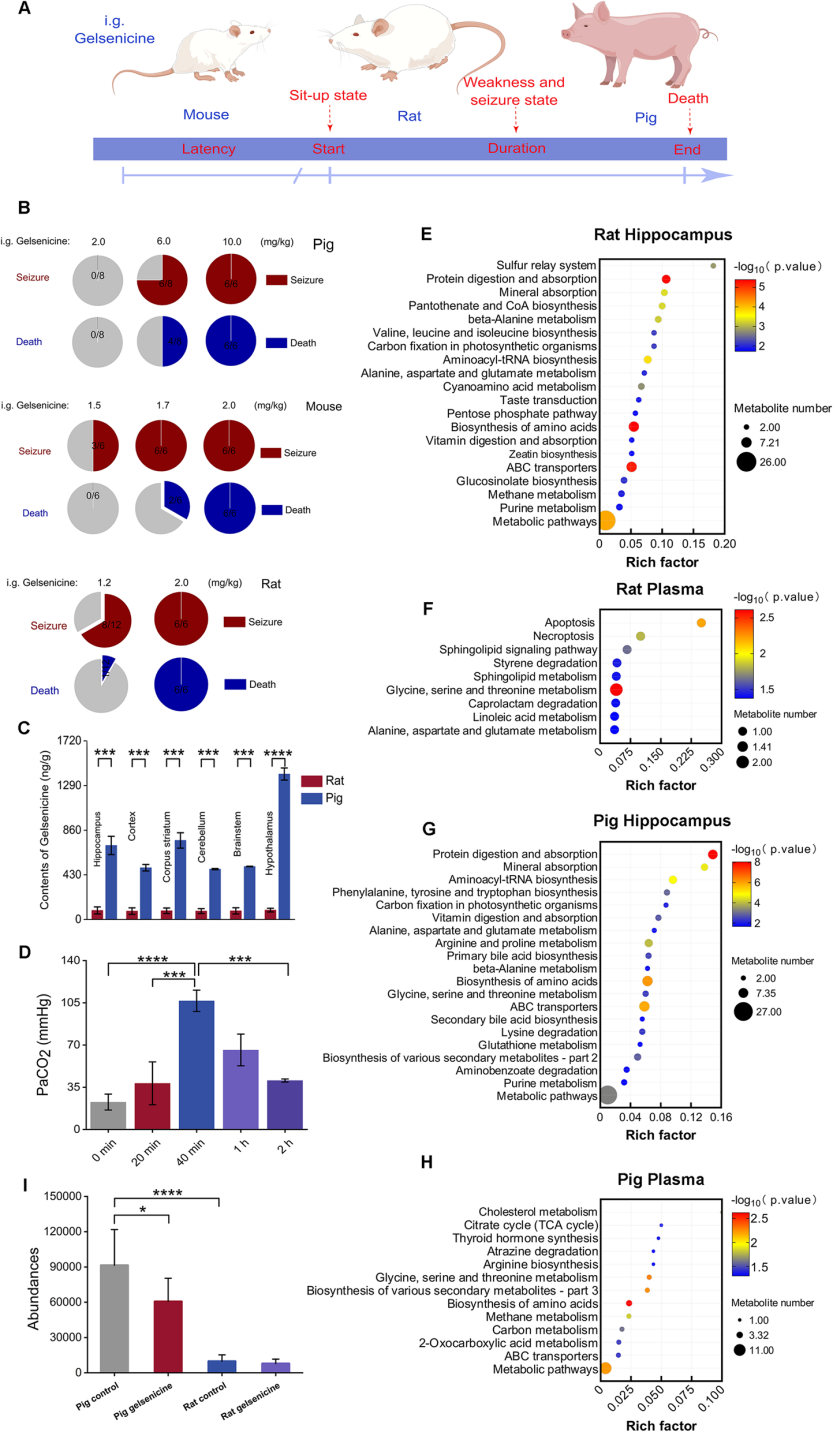
Variation in the toxicity of gelsenicine in mice, rats, and pigs and its relationship with circulating glycine levels (**A**) Schematic diagram of intragastric gavage (i.g.) of gelsenicine in mice, rats, and pigs. The figure was generated by Figdraw (www.figdraw.com). **B** Pie graphs depicting the toxicity and mortality caused by i.g. of gelsenicine at various doses from 2 to 10 mg/kg (*n* = 6–8 pigs, per group), 1.5 to 2 mg/kg (*n* = 6 mice, per group), and 1.2 to 2 mg/kg (*n* = 6–12 rats, per group). **C** Tissue distribution patterns of gelsenicine in pigs and rats after i.g. administration with gelsenicine at 2 mg/kg. *n* = 6–8, per group. **D** Arterial PaCO_2_ derived from pig arterial blood samples. *n* = 3 pigs. **E**–**H** Metabolomic profiling of the hippocampus and plasma in rat and pigs poisoned by gelsenicine. Each organ in the gelsenicine poisoning group was compared to the corresponding organ in the control group. Bubble diagram of KEGG enrichment pathway revealing significantly enriched KEGG pathways in the rat hippocampal region (**E**), rat plasma (**F**), pig hippocampal region (**G**), and pig plasma (**H**). The Y-axis represents the pathway name, and the X-axis represents the enrichment factor, that is the ratio of the number of differential metabolites enriched in the KEGG term to the total amount of differential metabolites. The greater the ratio, the greater the degree of enrichment. The size of the bubble indicates the number of genes in this pathway, and the shade of color depends on the *p*-value. *n* = 8, per group. **I** Changes in plasma glycine levels in pigs and rats. The y-axis represents the absolute values of peak areas of metabolites in the 100 µL plasma sample. *n* = 8, per group. Data are presented as mean ± SD. *P < 0.05, *** P < 0.001, ****P < 0.0001 based on one-way ANOVA or unpaired t tests

Previous studies have identified severe toxicity of *G. elegans* in humans and rats [34, 38], However, no reports of related poisoning in animals such as pigs and sheep have been documented [14, 15, 27]. We investigated potential species differences in the toxicity of gelsenicine by comparing the effects of gavage-administered gelsenicine in mice, rats, and pigs. Our results indicated that toxic doses of gelsenicine produced similar symptoms in mice and rats, implying no significant difference in toxicity between mice and rats (Fig. 2A, B). A low dose of gelsenicine was not toxic to pigs. Surprisingly, pigs exhibited an obvious toxic response to higher concentrations of gelsenicine. Although mice and rats (2 mg/kg) were more sensitive to gelsenicine toxicity than were pigs (6 mg/kg).

To understand the role of the tissue and blood concentrations of gelsenicine in the tolerance of pigs to toxicity, we used the test method for tissue concentration of gelsenicine developed by our team earlier to compare the gelsenicine distribution patterns in pig and rat tissues. The results (Additional file 2: Table S3–4) indicated that gelsenicine was distributed differently in pigs and rats at the same concentration of 2 mg/kg, with pigs having significantly higher concentrations in various brain regions, tissues, and organs than rats. Particularly, the concentrations were approximately 5–10 times higher in pigs than in rats (*P* < 0.01) (Fig. 2C). Despite the higher gelsenicine concentration in tissues, no discernible toxic response was observed in pigs, whereas rats experienced severe acute toxic reactions and died within approximately 20 min. At the time of death, the pigs had been administered a dosage of 10 mg/kg of gelsenicine, whereas the rats received a significantly lower dosage of only 2 mg/kg. The concentration of gelsenicine detected in the tissues and organs of pigs was approximately 20–30 times higher than that in rats, and the concentration in each brain region was 50–60 times higher. These findings indicate that neurotoxicity of gelsenicine is not strictly related to its ability to accumulate in the brain.

To further investigate the factors mediating differences in gelsenicine toxicity between pigs and mice, we selected the comparative analysis of arterial blood gas. The findings revealed that gelsenicine significantly inhibited respiratory function in both pigs and mice (Additional file 2: Tables S5–6). Interestingly, changes in the two groups of animals were similar. Following the administration of gelsenicine, both pigs and mice developed respiratory acidosis. The difference is that mice are more likely to experience a sharp increase in PaCO_2_ in a short time and induce death. Most pigs tolerated the hypoxic challenge and gradually recovered (Fig. 2D). These results suggest that pigs are more tolerant to hypoxia and hypercapnia, which is one of the factors contributing to the differences in toxicity of gelsenicine.

### A negative correlation between the circulating levels of glycine and the severity of gelsenicine poisoning

To investigate why pigs are more tolerant to hypoxia than rats, metabolomic analyses were conducted on the hippocampus and plasma samples from rats and pigs. The results revealed that the top three significant differences among the 34 differential metabolite enrichment pathways between rat control and gelsenicine-treated hippocampal samples were amino acid biosynthesis, protein digestion and absorption, and ABC transporter-related metabolic pathways (Fig. 2E). Metabolic pathway enrichment analysis of plasma samples from control and gelsenicine-treated rats revealed that glycine, serine, and threonine metabolism exhibited the most significant differences and enriched metabolites among the 14 differentially metabolite-enriched pathways, while the apoptotic metabolic pathways showed significant differences but fewer associated metabolites (Fig. 2F). The enrichment pathway analysis of 49 differential metabolites in pig hippocampal samples from the control and gelsenicine-treated groups demonstrated that protein digestion and absorption, amino acid biosynthesis, and ABC transporter-related metabolic pathways were the top three pathways with significant differences (Fig. 2G). Enrichment pathway analysis of 17 differential metabolites in the plasma samples of the pig control and gelsenicine-treated groups indicated that the top three pathways with significant differences were amino acid biosynthesis, glycine, serine, and threonine metabolism, and biosynthesis of various secondary metabolites (Fig. 2H). In summary, most of the effects of gelsenicine toxicity on endogenous metabolites in rats and pigs focused on amino acids, with glycine being the most important. Figure 2I illustrates that glycine concentrations in blank plasma varied significantly between species, with pigs being approximately 4–5 times higher than rats (*P* < 0.01). Based on the method developed for the precise quantification of neurotransmitters, the team previously examined glycine levels in the plasma of different animals, and the results revealed that glycine levels were significantly higher in pig plasma than in rats [39], consistent with the results of the present study. Furthermore, glycine levels in the hippocampus and several other brain regions of pigs are also higher than those in mice [40].

Previous studies have reported that glycine has a significant hypoxic-ischemic protective effect by enhancing the tolerance of the nervous system to hypoxia, thereby alleviating damage of hypoxia to the brain [41–43]. It has been proposed that higher levels of glycine in pigs may be one reason pigs are more tolerant to gelsenicine toxicity than rats and that glycine has a potential effect in improving hypoxia tolerance.

### Glycine rescued gelsenicine poisoning by hypoxia protection

**Fig. 3:**
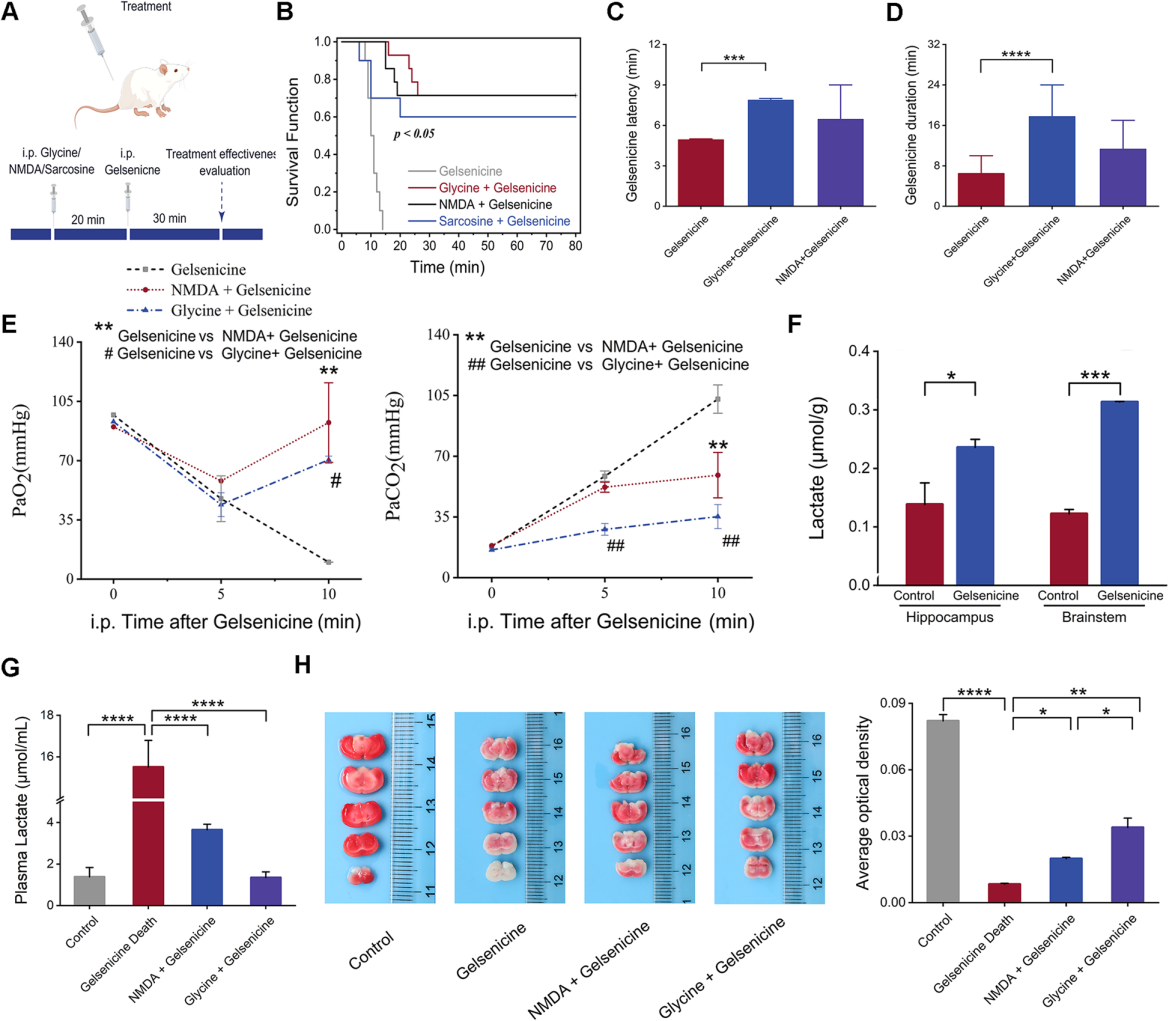
Glycine and NMDA in the therapeutic action of gelsenicine in ischemia-hypoxia reactions. **A** Schematic representation of the drug administration timeline. The figure was generated by Figdraw (www.figdraw.com). **B** Survival and protection curve of 0.24 mg/kg gelsenicine-infected mice pre-treated with 1600 mg/kg glycine, 25 mg/kg NMDA, and 800 mg/kg sarcosine. *n* = 14 mice/group. **C**–**E** Incubation period (**C**), toxic reaction time (**D**), and alterations in blood-gas parameters (**E**) after postoperative glycine and NMDA treatment. *n* = 3 mice/group. **F** Effects of i.p. injection of 0.24 mg/kg gelsenicine on the lactic acid content in the hippocampus and brain stem of mice. *n* = 3 mice/group. **G** Effects of i.p. injection of 0.24 mg/kg gelsenicine on the lactic acid content in the plasma of mice. *n* = 3 mice/group. **H** Representative brain sections for infarct size assessment by histological TTC staining and quantification of TTC-stained infarcts. *n* = 3 mice/group. Data are presented as mean ± SD. #/*P < 0.05, ##/**P < 0.01, ***P < 0.001, ****P < 0.0001 based on one-way ANOVA or unpaired t tests

To study whether high levels of glycine in pigs affected the toxicity of gelsenicine, mice were administered (i.p.) glycine, and gelsenicine 20 min later to determine whether glycine could resist the toxicity of gelsenicine (Fig. 3A). The results demonstrated that 0.24 mg/kg gelsenicine could induce acute poisoning in mice, resulting in a mortality rate of 100% (Fig. 3B). Survival curve analysis revealed that preventive administration of glycine significantly decreased the number of acute deaths caused by 0.24 mg/kg gelsenicine in mice (*P* < 0.05). Compared with the control group, the incubation period and poisoning time of mice in glycine + gelsenicine group were prolonged (Fig. 3C, D). It has been reported that sarcosine (a glycine transporter inhibitor) can increase the glycine concentration between synapses, which is then pre-rescued with sarcosine [44]. These results indicated that sarcosine also had a protective effect (Fig. 3B).

Previous studies have demonstrated that glycine protects against hypoxia by regulating the composition of the NMDAR subunits [45]. Interestingly, NMDA, a specific agonist of NMDARs, can rescue gelsenicine poisoning [13], but its protective mechanism remains unclear. One hypothesis is that NMDA could reduce gelsenicine toxicity by improving respiratory acidosis. To test this hypothesis, we evaluated the effects of glycine and NMDA on gelsenicine-induced respiratory inhibition. The results (Additional file 3: Table S7) revealed that NMDA and glycine effectively reversed the gelsenicine-induced decrease in PaO_2_ (*P* < 0.05) and increase in PaCO_2_ (*P* < 0.05) (Fig. 3E) and lactic acid (*P* < 0.05) (Fig. 3F, G), and fighting gelsenicine-induced hypoxia–ischemia (Fig. 3H; Additional file 4: Figure S1). Consistent with this, hyperventilation can relieve respiratory depression and reduce PaCO_2_, allowing cats to tolerate the toxicity of gelsemine (a gelsedine-type alkaloids) [46]. These results suggest that both NMDA and glycine exhibit neuroprotective during gelsenicine-induced hypoxia-ischemic and ameliorate respiratory depression. The difference in glycine content might be one of the reasons for the difference in the self-toxicity of gelsenicine between pigs and mice.

### The neurotoxicity of gelsenicine is related to GABA and glutamatergic pathways in hippocampus

**Fig. 4:**
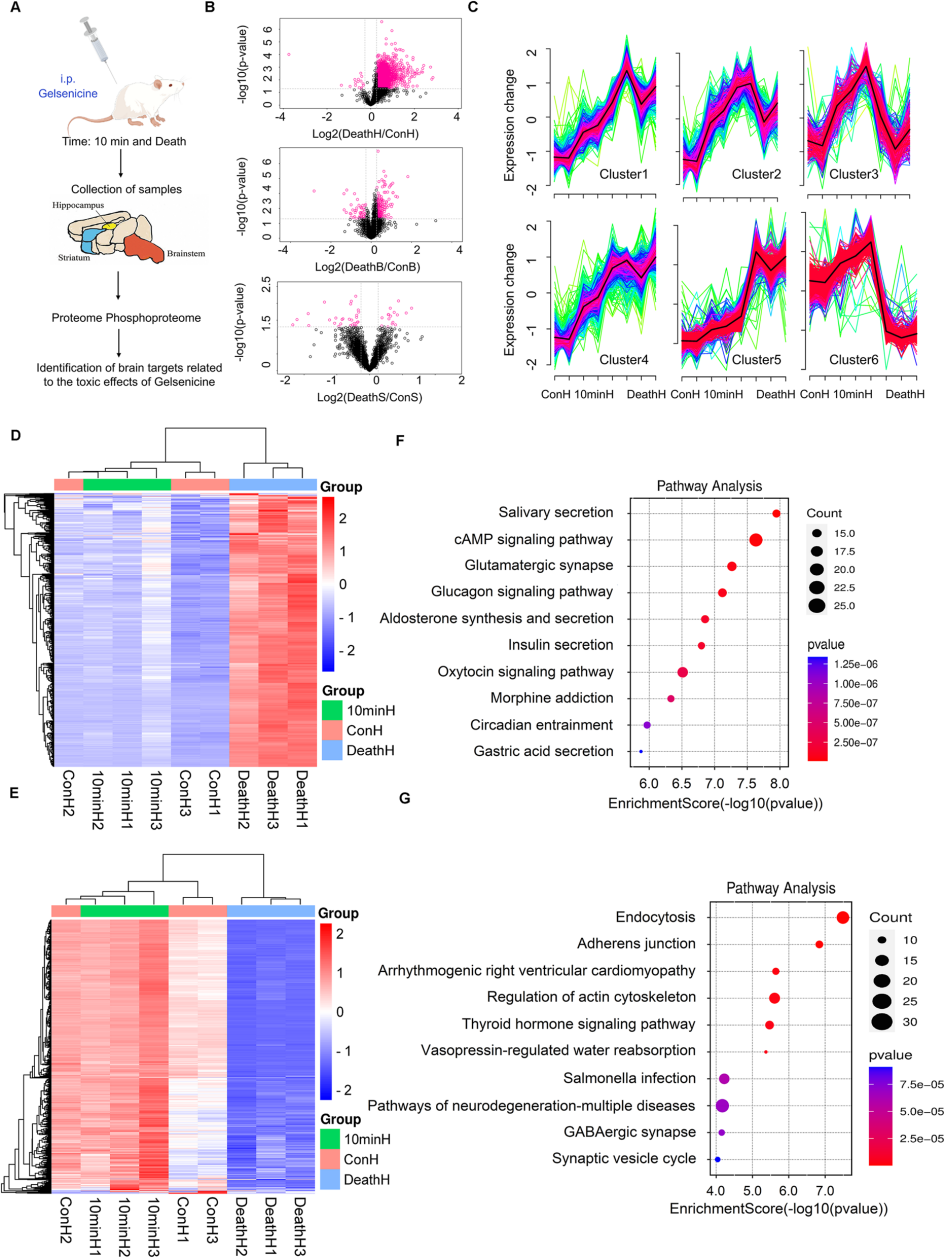
Phosphoproteomics at different time points in different brain regions of mice with gelsenicine poisoning. **A** A brief workflow of phosphoproteomics. Nine male mice were divided equally into the control, poisoning for 10 min, and death groups. Mice in the 10 min and death groups were i.p. injected with 0.2 mg/kg gelsenicine, while the control mice were treated with equal volumes of normal saline. All nine mice were euthanized. The hippocampus, brainstem, and striatum were extracted for phosphorylated proteomic analysis and data mining. *n* = 3 mice/group. **B** Volcano plots illustrating the differential phosphorylated proteins between the hippocampus (1st volcano plot), brainstem (2nd volcano plot), and striatum (3rd volcano plot) in the death group and the corresponding brain regions in the control group. **H**, hippocampus; B, brainstem. S, striatum. **C** Co-expression of the phosphorylated proteins in the hippocampus at different time points. **D**, **E** Clustering analysis by heatmap of phosphorylated proteins co-expressed in cluster 6 (**D**) and cluster 5 (**E**). The horizontal coordinate indicates different experimental groups (three-column replicates belong to one experimental group), and the vertical axis depicts the differentially phosphorylated proteins along with the results of hierarchical clustering. C1–3 denotes the control group; 10min1–3, the groups subjected to gelsenicine poisoning for 10 min; Death1–3, the groups corresponding to gelsenicine-induced death. **F**, **G** Significantly enriched KEGG pathways of phosphorylated proteins co-expressed in cluster 6 (**F**) and cluster 5(**G**)

**Fig. 5:**
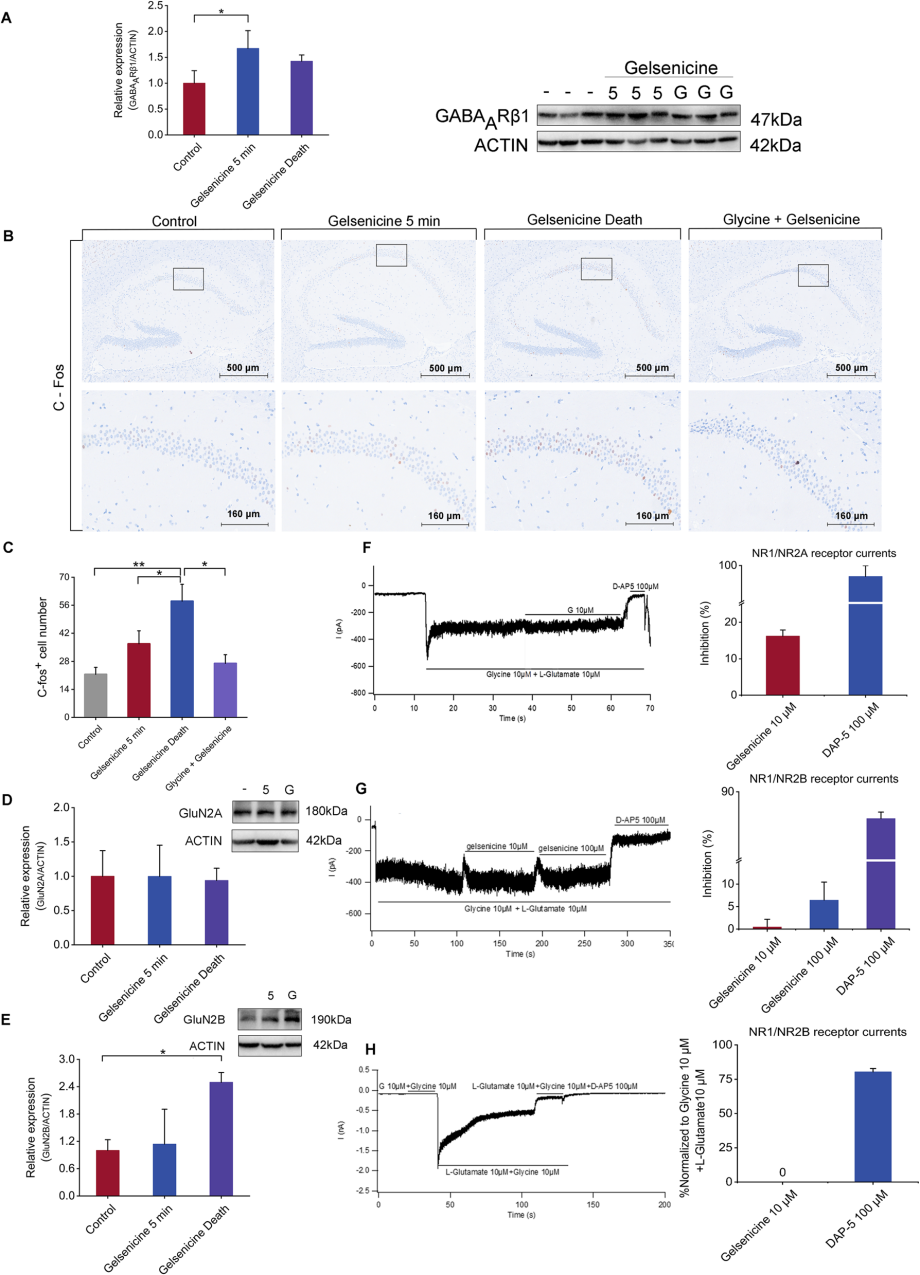
Induction of excitotoxicity through gelsenicine by hypoxia-induced disruption of NMDAR function. **A** GABA_A_Rβ1 protein expression in the hippocampus of mice. The graph represents the western blot quantification of the GABA_A_Rβ1/actin ratio. *n* = 3 mice/group. **B**, **C** Representative images of c-Fos expression in hippocampus after i.p. injection of 0.24 mg/kg gelsenicine (**B**). **C** For quantitative comparisons the total numbers of c-Fos-positive cells in the cellular layers of the hippocampus were counted. *n* = 3 mice/group. **D** Expression of GluN2A receptors in the hippocampus of mice. The graph represents Westen blot quantification of the GluN2A subunits/actin ratio. *n* = 3 mice/group. **E** Expression of GluN2B receptors in the hippocampus of mice. The graph represents the western blotting quantification of the GluN2B subunits/actin ratio. *n* = 3 mice/group. **F** Representative recording of the inhibitory effect of gelsenicine (10 μM) on GluN1/GluN2A receptor currents induced in HEK293 cells by application of 10 μM glutamate and 10 μM glycine. The graphs on the right show the values of the inhibition (in %) induced by Gelsenicine and DAP-5 on the NMDAR current responses; *n* = 2. **G** Representative recordings of the inhibiting effect of different gelsenicine concentrations (10 μM, 100 μM) on GluN1/GluN2B receptor currents. The graphs on the right show the values of the inhibition (in %) induced by Gelsenicine and DAP-5 on the NMDAR current responses; *n* = 2. **H** Representative recording of the effect of gelsenicine agonism on GluN1/GluN2B receptor currents. Galsenicine (10 μM) was pre-applied together with glycine (10 μM) for 20 s, followed by the application of of 10 μM glutamate and 10 μM glycine. The graphs on the right show the values of the potentiation (in %) induced by Gelsenicine and DAP-5 on the NMDAR current responses; *n* = 2. Mice in the 5 min and death groups received an i.p. injection of 0.24 mg/kg gelsenicine, control mice were injected with normal saline, and those in the Glycine + Gelsenicine groups were pre-treated with 1600 mg/kg glycine before receiving the same dose of gelsenicine. Data are presented as mean ± SD. *P < 0.05, **P < 0.01 based on one-way ANOVA

Gelsenicine has induced neurotoxicity. To identify the specific brain regions that are crucial to the toxic effect of gelsenicine, we performed proteomics of different brain regions in mice exposed to gelsenicine at various time points (Fig. 4A). Two brain regions, the hippocampus and brain stem, were activated during the poisoning process, as indicated by a significant increase in differentially phosphorylated peptide segments in these brain regions (Fig. 4B). However, only the phosphorylation level of the protein in the hippocampus increased continuously with time and reached a peak at death, indicating that the hippocampus plays a key role in the process of gelsenicine toxicity (Additional file 5: Table S8). Subsequently, to study the process of gelsenicine toxicity, we used time series analysis to demonstrate the dynamic changes in different proteins and pathways during the gelsenicine poisoning process. This allowed us to explore the biological characteristics of mice during different periods of gelsenicine poisoning. First, we focused on cluster 5 (Fig. 4C), which increased significantly in a time-dependent manner. This phosphoprotein may be associated with an increased severity of poisoning. This is particularly striking considering that cluster 6 phosphorylation expression varies significantly during different phases of poisoning, and it is significant for the blank control group, which aligns with the toxic attributes of the inhibition-excitation state transition in mice. Subsequently, we clustered the phosphorylated proteins expressed in clusters 6 (Fig. 4D) and 5 (Fig. 4E) by layer and analyzed the enrichment of the KEGG pathway. Phosphorylated proteins in glutamatergic synaptic pathways were significantly upregulated with time (Fig. 4F). Phosphorylated proteins in the GABAergic synaptic pathway in the hippocampus were upregulated for 10 min (Fig. 4G). In contrast, those in the death group were significantly downregulated. These results indicate that the GABAergic nerve pathway may be involved in the early inhibitory state of gelsenicine-poisoned mice and that the increasing degree of spasm in mice is related to the increased excitability of glutamate.

### Gelsenicine induced respiratory depression by regulating the function of GABA_A_R

GABAR has been reported to be closely associated with respiratory disorders [47, 48]. Persistent activation of GABA_A_R may result in respiratory depression, leading to a severe hypoxia–ischemia response. Our findings indicate that the toxicity of gelsenicine is closely associated with GABA_A_R. On one hand, previous electrophysiological data demonstrated that gelsenicine could regulate GABA_A_R and significantly prolong the opening time of *chloride* channels. On the other hand, western blotting revealed that GABA_A_Rβ1 protein was explicitly overexpressed in the hippocampus of mice poisoned with 0.24 mg/kg gelsenicine for 5 min (*P* < 0.05) (Fig. 5A). These results suggest that gelsenicine inhibits the central nervous system (CNS) and induces respiratory depression through GABA_A_R.

### Gelsenicine caused hypoxia to induce NMDAR overexcitation

Due to the convulsions observed in mice treated with gelsenicine, we examined c-Fos expression (Fig. 5B, C), a direct marker of neuronal activation [49], to verify the effect of gelsenicine on the excitability of brain neurons. We observed that gelsenicine triggered extensive neuronal activation in the hippocampus. These results suggested that the excitotoxicity is involved in the toxicity of gelsenicine.

NMDARs, a subtype of ionic glutamate receptors, play a vital role in excitatory toxicity [50, 51]. The NMDAR is composed of four subunits, of which NMDAR 1, 2 (A–D) and 3 (A and B) assemble to form a tetrameric structure, while GluN1 is the necessary subunit, and GluN2 (A–D) and GluN3 (A and B) are the regulatory subunits, of which GluN2 subunit is the most important one [52]. Most NMDARs have two GluN1 and two GluN2A or GluN2B subunits. We explored the effect of gelsenicine on NMDAR protein expression using protein blot analysis. There was no difference in the expression of GluN2A receptors at different times of poisoning in mice (Fig. 5D). In contrast, gelsenicine significantly increased GluN2B receptors expression in the hippocampus (*P* < 0.05) (Fig. 5E). These effects, combined with the rescue effect of NMDA for gelsenicine poisoning, indicate that NMDARs are involved in the neuroexcitotoxicity of gelsenicine. However, gelsenicine must directly or indirectly affect the NMDAR to induce NMDAR-related excitotoxicity.

To investigate the interaction between gelsenicine and NMDARs, we assessed the effects of gelsenicine on NMDAR function in HEK-293 cells using patch-clamp recordings. Our results demonstrated that 10 µM gelsenicine did not significantly inhibit GluN2A or GluN2B channels (Fig. 5F, G), and did not stimulate NMDAR currents in GluN2B receptors-expressing HEK-293 cells (Fig. 5H). These findings revealed that the effect of gelsenicine on NMDARs is not direct excitation or inhibition. This effect can potentially be indirect. For example, glutamatergic excitotoxicity is a common factor in acute events like cerebral hypoxic-ischemia. The respiratory depression observed in mice poisoned with gelsenicine could potentially result in NMDAR-mediated excitotoxicity [53, 54].

### Difference of NMDAR content and tolerance to the toxicity of gelsenicine

**Fig. 6:**
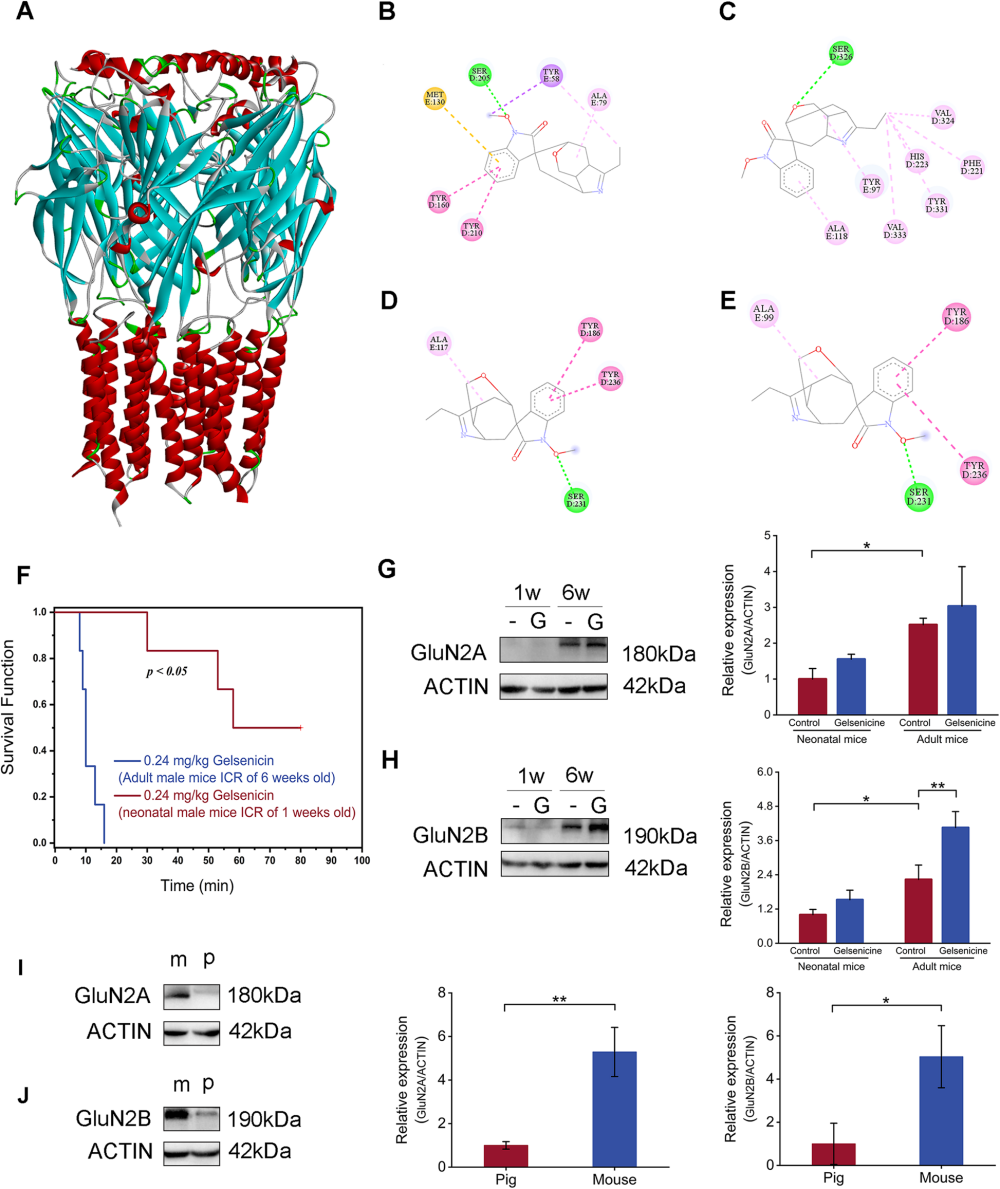
Differences in NMDAR levels and tolerance to gelsenicine toxicity. **A**–**E** Three-dimensional structure **A** of pig GABA_A_R constructed by homologous modeling and the two-dimensional diagram of the binding mode of gelsenicine with human (**B**), pig (**C**), rat (**D**), and mouse GABA_A_R (**E**). The purple dotted line denotes the pi-sigma interaction, the dark pink dotted line denotes the pi-pi stacking interaction, the light pink dotted line represents the alkyl interaction, the yellow dotted line denotes the pi-sulfenyl interaction, and the green dotted line indicates the hydrogen bonding interaction. **F** Survival and protection curves for gelsenicine application in newborn (1 week old) and adult (6 weeks old) mice. *n* = 6 mice/group. **G** Expression of GluN2A subunits in the hippocampus of mice. The graph represents the western blot quantification of the GluN2A subunit/actin ratio. Mice in the gelsenicine group were intraperitoneally injected with 0.24 mg/kg gelsenicine, while the control mice were treated with equal volumes of normal saline. 1w stands for one week old, and 6w stands for six weeks. *n* = 3 mice/group. **H** Expression of GluN2B subunits in the hippocampus of mice. The graph represents the western blot quantification of the GluN2B subunits /actin ratio. *n* = 3 mice/group. **I**, **J** Expression of GluN2A (**I**) and GluN2B (**J**) subunits in the hippocampus of adult mice and pigs. Both mouse and pig samples were used as blanks. The graphs represent the western blot quantification of the GluN2 subunits/actin ratio. *n* = 3 mice/group Data are presented as mean ± SD. *P < 0.05, **P < 0.01 based on one-way ANOVA or unpaired t tests

These results suggest that early respiratory depression may involve the GABAergic nerve pathways. To investigate the potential correlation between the difference in toxicity of gelsenicine and its relative affinity for different GABAR species, we conducted molecular docking research based on GABA_A_R models of different species. Currently, because the crystal structure of pig-derived GABA_A_R has not been solved, we obtained the structures of pig-derived, rat-derived, and mouse-derived GABA_A_R using the protein crystal structure of human-derived GABA_A_R [55] as a template for homologous modeling (Figs. 6A–E). Molecular docking revealed that the binding free energies of gelsenicine with human, pig, rat, and mouse GABA_A_R were –7.76, –8.81, –7.80, and –7.79 kcal/mol, respectively. All these values were less than –7.0 kcal/mol, indicating that gelsenicine has a strong binding affinity with different species of GABA_A_R. In addition to hydrophobic interactions, such as alkyl interactions and Pi-Pi stacking, gelsenicine consistently formed hydrogen bonds with different species of GABA_A_R. These data suggest that hydrophobic interactions and hydrogen bond formation may be the main interactions that help gelsenicine bind to GABA_A_R. However, the variations in binding modes and energies between gelsenicine and GABA_A_Rs across species did not exhibit a clear correlation with toxicity, indicating that the differential toxicity of gelsenicine among species is not solely attributable to the binding affinity differences with GABA_A_Rs.

Previous studies have indicated that neonatal mice are more resistant to hypoxia than adult mice [56, 57]. To further explore the relationship between gelsenicine toxicity and its tolerance to natural hypoxia, we conducted a study on the developmental toxicity of gelsenicine. The results revealed that neonatal mice had higher tolerance to gelsenicine (Fig. 6F). Most neonatal mice lived for at least 40 min, while all adult mice died within 10–20 min. These observations were consistent with the difference in the toxicity of gelsenicine between pigs and mice.

Notably, some known hypoxia-tolerant animals, including sea turtles, 13-lined ground (13LG) squirrels, and naked moles, could significantly altering the subunit composition of NMDAR by reducing GluN1/GluN2A and GluN1/GluN2B during hypoxia to avoid a series of harmful events caused by hypoxia-induced NMDAR overexcitation [58]. To investigate the mechanisms underlying the tolerance of neonatal rats and pigs to brain hypoxia, we measured NMDAR levels in the brains of neonatal rats, adult rats, and pigs. Compared to neonatal rats and pigs, adult mice had more NMDAR proteins (*P* < 0.05) (Fig. 6G–J). In summary, our results demonstrate that low levels of NMDARs in the hippocampus can resist gelsenicine-induced NMDAR overexcitation.

### Gelsenicine caused mitochondrial energy metabolism disorder and Ca^2+^ overload

**Fig. 7:**
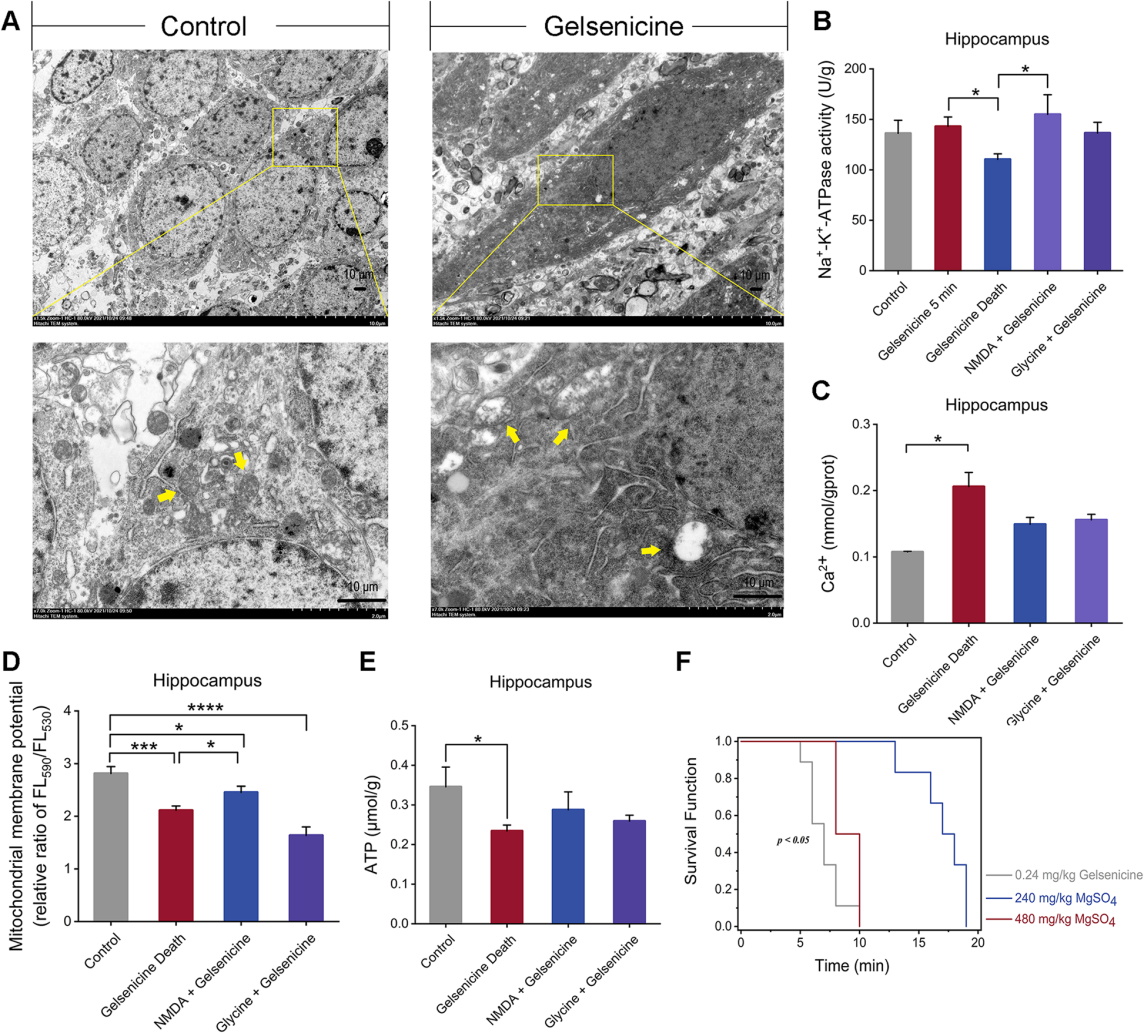
Gelsenicine-mediated mitochondrial toxicity. **A** TEM of gelsenicine-induced mitochondrial damage in the hippocampus. The yellow arrows indicate mitochondria. *n* = 3 mice/group. **B**–**E** Na^+^-K^+^-ATPase activity (**B**), Ca^2+^ concentration (**C**), mitochondrial membrane potential (**D**), and ATP content (**E**) in the hippocampus of mice treated with 0.24 mg/kg gelsenicine. The mitochondrial membrane potential was measured by a fluorescent enzyme analyzer. The ratio of fluorescence intensities Ex/Em = 525/590 nm and 490/530 nm (FL590/FL530) were recorded to delineate the mitochondrial membrane potential level of each sample. *n* = 3 mice/group. **F** Survival and protection curve of 0.24 mg/kg gelsenicine-infected mice pre-treated with MgSO_4_. *n* = 6 mice/group Mice in the 5 min and death groups received an i.p. injection of 0.24 mg/kg gelsenicine, control mice were injected with normal saline. Mice in the Glycine/NMDA + Gelsenicine groups were pre-treated with either 1600 mg/kg glycine or 25 mg/kg NMDA, respectively, prior to receiving the identical dose of 0.24 mg/kg gelsenicine. Data are presented as mean ± SD. *P < 0.05, *** P < 0.001, ****P < 0.0001 based on one-way ANOVA or unpaired t tests

Mitochondrial toxicity is also the main death pathway in gelsenicine-induced neuroexcitatory toxicity injury after hypoxia–ischemia. First, we performed TEM to investigate the effects of gelsenicine on mitochondrial energy metabolism. Our findings indicate that gelsenicine caused damage to the mitochondria of the mouse hippocampus (Fig. 7A). Specifically, the mitochondria exhibited moderate swelling, shallow dissolution of the matrix, and disorganized arrangement, with most of the cristae broken or disappeared. Consistent with TEM results, the acute toxic dosage of gelsenicine resulted in a reduction of Na^+^-K^+^-ATPase activity (*P* < 0.05) (Fig. 7B), and concurrently elevated the overall concentration of free Ca^2+^ in the hippocampal tissue (*P* < 0.05) (Fig. 7C). Furthermore, it is characterized by a decline in the mitochondrial membrane potential (*P* < 0.05) (Fig. 7D) and a reduction in ATP content (*P* < 0.05) (Fig. 7E), indicating mitochondrial-dependent toxicity. These findings suggest that the integrity of mitochondrial function is compromised and that the mitochondria contribute to the neurotoxic effects of gelsenicine.

Excessive activation of NMDARs and mitochondrial energy dysfunction triggered by gelsenicine ultimately result in Ca^2+^ overload, which plays a critical role in the neurotoxicity effects of the gelsenicine. Consequently, one potential strategy to mitigate gelsenicine toxicity is to counteract the Ca^2+^ overload in neurons. We performed a rescue study of gelsenicine poisoning using anticonvulsant pretreatment. A low dose of magnesium sulfate (a calcium channel blocker) administered i.p. significantly prolonged the time of death in mice (*P* < 0.05) (Fig. 7F). Notably, mice that received a high dose of magnesium sulfate did not experience convulsions during the time of death; however, the time of death was not affected. These findings suggest that a low concentration of magnesium sulfate may prevent the convulsive effects of gelsenicine, delaying the onset, attack time, and peak value of tremors. High doses of magnesium sulfate can lead to magnesium poisoning because of its narrow safety range. This could inhibit the respiratory center in the medulla oblongata, thereby exacerbating the respiratory inhibition toxicity caused by gelsenicine, ultimately leading to the death of the mice. Our results suggested that the severe convulsive effects observed in gelsenicine poisoning may be related to Ca^2+^ overload. Although combating Ca^2+^ overload can alleviate acute convulsive symptoms, it does not reduce mortality.

## Discussion

Currently, more than 120 alkaloids have been identified in *G. elegans*, grouped into six types based on their chemical structures: sarpagine-type, gelsemine-type, gelsedine-type, humantenine-type, koumine-type and yohimbane-type [1]. According to the research findings on the acute toxicity of *G. elegans* extracts and alkaloids in rats and mice, it is evident that there is significant variation in toxicity among different types of Gelsemium alkaloids. In mice, LD_50_ for gelsemine-type alkaloids administered intravenously (i.v.) is 78.23 mg/kg [59]; for koumine-type alkaloids administered i.p. the LD_50_ value exceeds 50 mg/kg [60]. However, gelsenicine is a key compound in gelsedine-type alkaloids, with an LD_50_ of just 0.185 mg/kg following i.p. injection in mice [13]. In addition, the LD_50_ of the *G. elegans* extract is between 1.5–15 mg/kg [59]. Recent studies believe that gelsenicine serve as the predominant toxic constituent of *G. elegans* due to its high toxicity and toxicological profile, which closely mirrors that of the *G. elegans* extract [13, 21].

Recent research has indicated that GABAR is important in regulating respiratory rhythms. For example, propofol (a GABA_A_R agonist) induces severe respiratory depression [61–63], which is partially attributed to GABA_A_R stimulation [64, 65]. Because of the increase in Cl^−^ influx and prolongation of postsynaptic current inhibition, propofol induces cyanosis-like symptoms, manifesting as hypoventilation and increased arterial CO_2_ levels [66, 67]. Our team has consistently proved that gelsenicine does not directly activate GABA_A_R, but enhance interaction of GABA with GABA_A_R and prolong the opening time of Cl^−^ channels, resulting in its effects on neurons in the CNS; Moreover, gelsenicine (300 µM) has little effect on evoked currents mediated by glycine and nicotinic acetylcholine receptors [68]. Subsequent research has demonstrated that GABA_A_R antagonists, flumazenil and picrotoxin, can effectively decrease mortality in mice poisoned by 14-(R)-hydroxy-gelsenicine [22]. In agreement with this concept, flumazenil, which demonstrates greater binding energy to GABA_A_R than gelsenicine, can play a role in rescuing the toxicity of gelsenicine by antagonize its effect on GABA_A_Rs [23, 24]. Therefore, gelsenicine-induced respiratory inhibition of the CNS is primarily mediated by enhancing the effect of GABA on GABA_A_R. This hypothesis has been proven by the experimental results of the present study. Gelsenicine binds to GABA_A_R by forming hydrophobic and hydrogen bonds.

There is increasing evidence that hypoxia–ischemia triggers a cascade of neurotoxic events, which contribute to activation of NMDARs that are responsible for excitotoxicity [69–71]. Gelsenicine consistently induces excitotoxicity; although gelsenicine does not directly activate or inhibit NMDARs, the excitotoxicity related to NMDARs after hypoxia–ischemia mediated by GABAR remains a pivotal toxic process, based on our findings from TTC staining, the c-Fos staining, electrophysiological, phosphoproteomics and behavioral studies. Accordingly, the present study provides insights into the species-specific toxicity of gelsenicine. Low levels of NMDARs in the hippocampus could resist gelsenicine-induced NMDAR overexcitation.

As an agonist of the NMDAR, NMDA pretreatment can mitigate the toxicity of gelsenicine, which may be related to the establishment of hypoxic tolerance after cerebral hypoxic-ischemic preconditioning. Numerous studies have demonstrated that non-convulsive doses of NMDA preconditioning can mitigate seizures linked to NMDAR excitotoxicity. Direct stimulation of NMDARs with low concentrations of NMDA, followed by desensitization, provides significant neuroprotection against subsequent severe hypoxic-ischemic excitotoxic injury [51, 72–75]. Additionally, it is well-established that anesthetics can exhibit neuroprotective effects against their own neurotoxic potential. This is evidenced by the capacity of prior low-dose anesthetic exposure to significantly reduce brain damage caused by high-dose or prolonged anesthetic exposure, as observed with isoflurane [76].

Our study found that pigs have a much higher tolerance to gelsenicine-induced toxicity than other species. A significant difference between pigs and humans or mice is the level of circulating glycine. Circulating glycine levels in humans [77, 78], rats and mice [79, 80] range from 0.2–0.4 mM, whereas 1–1.5 mM in pigs [81]. This study demonstrated that glycine could treat the toxicity of gelsenicine, indicating that differences in circulating glycine levels may be one of the reasons for this difference in species-specific toxicity. To our knowledge, this is the first study to systematically examine the mechanisms underlying the high tolerance of pigs to gelsenicine-induced toxicity.

This study had certain limitations. While we have demonstrated that the neurotoxic effects of gelsenicine can be attributed to mechanisms involving GABARs, future in vivo investigations utilizing GABARs gene knockout mice are necessary to fully understand the pivotal role of GABARs in gelsenicine-induced neurotoxicity. In addition, primary CNS neuronal cultures to investigate the impact of gelsenicine on nerve cells and receptor balance through electrophysiological experiments. This will help to explore intracellular signal transduction pathways and alterations in key proteins.

## Conclusions

The findings illustrated the important contribution of GABAR to Cl^−^ channel change, which is essential for the respiratory inhibition by gelsenicine, thereby leading to overexcitation of the NMDARs and mitochondrial dysfunction. This study deciphered the toxic process of gelsenicine and identify a potential therapeutic drug, glycine, which could provide theoretical basis and data support for guiding the clinical treatment of *G. elegans*-induced toxicity. However, the underlying mechanisms involved in the neuroprotective effects of NMDA and glycine must be further investigated to delineate a better understanding of the relationship between *G. elegans*-induced neurotoxicity and hypoxia tolerance evoked by NMDA and glycine preconditioning.

## Supplementary Information

Additional file 1: Table S1-S2. Effects of gelsenicine on the physiological indices of ICR mice. Table S1. Alterations in blood gas parameters after intraperitoneal administration of gelsenicine to ICR mice (average x±s). Table S2. The blood routine parameters of the mouse treated with 0.24mg/kg gelsenicine for 10 min. Gelsenicine has significantly induced respiratory depression. pH and PaO_2_ in the 0.24 mg/kg gelsenicine group were significantly lower than those in the blank control group (*P* < 0.05), while PaCO_2_, HCO_3_ and K^+^ were significantly increased as compared with those in the blank control group (*P* < 0.05). The values are expressed as the mean ± SD (*n *= 3 per group). Abbreviations: Pa, partial pressure; Hct, hematocrits; AG, anion gap. WBC (white blood cell count), LY (lymphocyte), MO (monocytes), NE (neutrophilic granulocyte), RBC (red blood cell count), HGB (hemoglobin concentration), HCT (hematocrits), MCV (mean corpuscular volume), MCH (mean corpuscular hemoglobin), MCHC (mean corpuscular hemoglobin concentration), RDW (red cell distribution width), PLT (platelet count), MPV (mean platelet volume), PDW (platelet distribution width), PCT (plateletcrit).

Additional file 2: Table S3-S6. Gelsenicine administration in pigs and rats for determination of drug concentrations in plasma and tissues, and for arterial blood gas analysis. Table S3. Distribution of gelsenicine at different concentrations in the tissues and plasma of pigs and rats. Table S4. Distribution of different concentrations of gelsenicine in various brain regions and spinal cords of pigs and rats. The time to death of rats treated with 2 mg/kg of gelsenicine and pigs treated with 10 mg/kg of gelsenicine was approximately 15–30 min. *n* = 3–8, per group. Table S5. Effect of 2 mg/kg gelsenicine on blood gas parameters in mice. Table S6. Effects of 6 mg/kg gelsenicine by gavage on blood gas parameters of pigs. *n* = 3 pigs.

Additional file 3: Table S7. Effect of glycine and NMDA on respiratory inhibition induced by gelsenicine. *n*=3 mice/group.

Additional file 4: Figure S1. Representative heart sections for infarct size assessment by histological TTC staining and quantification of TTC-stained infarcts. Gelsenicine increased myocardial hypoxia-ischemia injury in the mice. NMDA and glycine preconditioning attenuated the damage induced by gelsenicine and improved cardiac function. Mice in gelsenicine groups received an i.p. injection of 0.24 mg/kg gelsenicine, control mice were injected with normal saline. Mice in the Glycine/NMDA + Gelsenicine groups were pre-treated with either 1600 mg/kg glycine or 25 mg/kg NMDA, respectively, prior to receiving the identical dose of 0.24 mg/kg gelsenicine. Data are represented as mean ± SD. *** *P* < 0.001, ****P < 0.0001 based on one-way ANOVA or unpaired t tests. *n*=3 mice/group.

Additional file 5: Table S8. Statistical analysis of changes in phosphorylated peptides. Mice in the 10 min and death group were intraperitoneally injected with 0.2 mg/kg gelsenicine, while the control mice were treated with equal volumes of normal saline. All the nine mice were euthanized. Abbreviations: H, hippocampus; B, brainstem. S, striatum.

Additional file 6. The original, uncropped gels.

Additional file 7. The Arrive checklist.

## Data Availability

No datasets were generated or analysed during the current study.

## Abbreviations

AG: Anion gap
BBB: Blood-brain barrier
CAMERA: Collection of Algorithms of MEtabolite pRofile Annotation
CNS: Central nervous system
DMEM: Dulbeccos modified Eagles medium
DAP-5: D(-)-2-Amino-5-phosphonovaleric acid
DAB: Diaminobenzidine
*G. elegans*: *Gelsemium elegans*
GABA: Gamma-aminobutyric acid
GABARs: GABA receptors
HRP: Horseradish Peroxidase
LC–MS/MS: Liquid chromatography-tandem mass spectrometry
NMDA: N-methyl-D-aspartate
NMDARs: NMDA receptors
P-gp: P-glycoprotein
SD: Standard deviation
TTC: 2,3,5-Triphenyltetrazolium chloride
TEM: Transmission electron microscopy
2D-LC-UV: Two-dimensional liquid chromatography with Ultraviolet detection
