# Supplementary material for: Hypoxia tolerance determine differential gelsenicine-induced neurotoxicity between pig and mouse
Source: BMC Med. 2025 Mar 12;23:156. doi: 10.1186/s12916-025-03984-5 (PMC11905507; doi:10.1186/s12916-025-03984-5)
Supplement: Supplementary file 1 — Additional file 1: Table S1-S2. Effects of gelsenicine on the physiological indices of ICR mice. Table S1. Alterations in blood gas parameters after intraperitoneal administration of gelsenicine to ICR mice (average x±s). Table S2. The blood routine parameters of the mouse treated with 0.24mg/kg gelsenicine for 10 min. Gelsenicine has significantly induced respiratory depression. pH and PaO2 in the 0.24 mg/kg gelsenicine group were significantly lower than those in the blank control group (P < 0.05), while PaCO2, HCO3 and K+ were significantly increased as compared with those in the blank control group (P < 0.05). The values are expressed as the mean ± SD (n = 3 per group). Abbreviations: Pa, partial pressure; Hct, hematocrits; AG, anion gap. WBC (white blood cell count), LY (lymphocyte), MO (monocytes), NE (neutrophilic granulocyte), RBC (red blood cell count), HGB (hemoglobin concentration), HCT (hematocrits), MCV (mean corpuscular volume), MCH (mean corpuscular hemoglobin), MCHC (mean corpuscular hemoglobin concentration), RDW (red cell distribution width), PLT (platelet count), MPV (mean platelet volume), PDW (platelet distribution width), PCT (plateletcrit). [file 12916_2025_3984_MOESM1_ESM.docx]

**Table S1**

Alterations in blood gas parameters after intraperitoneal administration of gelsenicine to ICR mice (average‾x±s).

| Test items | basic line | 0.24 mg/kg Gelsenicine | | 0.12 mg/kg Gelsenicine | | |
| --- | --- | --- | --- | --- | --- | --- |
|  |  | 5 min | 10 min | 5 min | 15 min | 30 min |
| PaO_2_ (mmHg) | 91.00±18.52 | 47.50±13.50 | 10.00±0.10 | 82.50±7.50 | 41.75±14.24 | 89.00±13.86 |
| PaCO_2_ (mmHg) | 18.45±1.48 | 58.50±30.00 | 102.93±8.16 | 40.60±0.30 | 49.70±9.58 | 49.30±7.35 |
| Hct (%) | 37.33±1.15 | 35.50±2.50 | 37.00±12.29 | 29.50±3.50 | 35.00±4.40 | 41.00±6.38 |
| pH | 7.33±0.04 | 7.17±0.01 | 6.86±0.07 | 7.27±0.01 | 7.24±0.07 | 7.21±0.09 |
| Na^+^ (mmol/L) | 150.30±2.52 | 148±0.00 | 155.00±1.73 | 148.00±1.94 | 150.00±3.83 | 153.00±1.41 |
| K^+^ (mmol/L) | 4.63±0.21 | 5.75±0.45 | 6.55±0.21 | 4.35±0.05 | 4.93±0.22 | 4.30±1.29 |
| Ca^2+^ (mmol/L) | 1.32±0.20 | 1.28±0.02 | 1.32±0.05 | 1.16±0.07 | 1.30±0.11 | 1.31±0.24 |
| Cl^-^ (mmol/L) | 124.00±4.24 | 119.50±2.54 | 122.67±3.51 | 116.00±4.65 | 117.00±6.27 | 121.00±5.30 |
| HCO_3_^-^ (mmol/L) | 13.23±1.91 | 20.85±0.55 | 17.93±2.21 | 18.05±0.45 | 20.78±3.04 | 19.30±2.20 |
| AG (mmol/L) | 16.50±3.54 | 13.50±2.50 | 22.33±2.52 | 18.50±2.54 | 17.50±3.42 | 17.00±2.56 |

Note: Gelsenicine has significantly induced respiratory depression. pH and PaO_2_ in the 0.24 mg/kg gelsenicine group were significantly lower than those in the blank control group (*P* < 0.05), while PaCO_2_, HCO_3_^-^ and K^+^ were significantly increased as compared with those in the blank control group (*P* < 0.05). Pa, partial pressure; Hct, hematocrits; AG, anion gap. n=3 mice/group.

**Table S2**

The blood routine parameters of the mouse treated with 0.24 mg/kg gelsenicine for 10 min.

| Test items | Normal control group | 0.24 mg/kg Gelsenicine |
| --- | --- | --- |
|  | n = 4 | n = 4 |
| WBC (10^9^/L) | 9.80±1.01 | 9.60±0.42 |
| LY (10^9^/L) | 7.07±0.90 | 7.25±0.92 |
| MO (10^9^/L) | 0.25±0.38 | 0.25±0.07 |
| NE (10^9^/L) | 1.65±1.12 | 1.80±0.42 |
| LY% (%) | 78.60±11.98 | 74.90±6.51 |
| MO% (%) | 3.05±2.87 | 3.20±0.57 |
| NE% (%) | 22.97±9.15 | 21.90±5.94 |
| RBC (10^12^/L) | 10.41±0.50 | 10.51±0.30 |
| HGB (g/L) | 150.00±4.58 | 160.67±11.02 |
| HCT (%) | 49.47±1.93 | 53.13±3.93 |
| MCV (fL) | 47.60±1.55 | 50.57±2.41 |
| MCH (pg) | 14.40±0.53 | 15.23±0.64 |
| MCHC (g/L) | 302.67±2.89 | 301.67±3.21 |
| RDW (%) | 15.57±0.38 | 14.97±1.33 |
| PLT (10^9^/L) | 511.33±124.42 | 311.00±417.19 |
| MPV (fL) | 5.93±0.39 | 5.60±0.30 |
| PDW | 16.93±0.57 | 17.23±0.06 |
| PCT (%) | 0.30±0.05 | 0.18±0.23 |

Note: The values are expressed as the mean ± SD (n = 3 per group)

Abbreviations: WBC (white blood cell count), LY (lymphocyte), MO (monocytes), NE (neutrophilic granulocyte), RBC (red blood cell count), HGB (hemoglobin concentration), HCT (hematocrits), MCV (mean corpuscular volume), MCH (mean corpuscular hemoglobin), MCHC (mean corpuscular hemoglobin concentration), RDW (red cell distribution width), PLT (platelet count), MPV (mean platelet volume), PDW (platelet distribution width), PCT (plateletcrit).
