# Supplementary material for: Hypoxia tolerance determine differential gelsenicine-induced neurotoxicity between pig and mouse
Source: BMC Med. 2025 Mar 12;23:156. doi: 10.1186/s12916-025-03984-5 (PMC11905507; doi:10.1186/s12916-025-03984-5)
Supplement: Supplementary file 2 — Additional file 2: Table S3-S6. Gelsenicine administration in pigs and rats for determination of drug concentrations in plasma and tissues, and for arterial blood gas analysis. Table S3. Distribution of gelsenicine at different concentrations in the tissues and plasma of pigs and rats. Table S4. Distribution of different concentrations of gelsenicine in various brain regions and spinal cords of pigs and rats. The time to death of rats treated with 2 mg/kg of gelsenicine and pigs treated with 10 mg/kg of gelsenicine was approximately 15–30 min. n = 3–8, per group. Table S5. Effect of 2 mg/kg gelsenicine on blood gas parameters in mice. Table S6. Effects of 6 mg/kg gelsenicine by gavage on blood gas parameters of pigs. n = 3 pigs. [file 12916_2025_3984_MOESM2_ESM.docx]

**Table S3**

Distribution of gelsenicine at different concentrations in the tissues and plasma of pigs and rats.

| Animals | Dose  (mg/kg) | Sampling time | Tissue concentration of gelsenicine (ng/g) | | | | | | | | |
| --- | --- | --- | --- | --- | --- | --- | --- | --- | --- | --- | --- |
|  |  |  | Heart | Liver | Spleen | Lung | Kidney | Muscle | Intestines | Faeces | Plasma |
| Pigs | 10 | Dead time | 2851.69±575.59 | 4929.78±193.88 | 5333.63±487.78 | 6580.38±619.43 | 7173.53±403.28 | 3342.73±561.43 | 5004.64±389.49 | 159.04±67.66 | 709.90±276.84 |
| Pigs | 6 | 20 min | 429.89±134.34 | 1608.59±693.15 | 961.37±244.47 | 1673.78±338.83 | 2746.95±560.40 | 715.38±263.01 | 1072.15±122.10 | 349.44±12.22 | 551.80±616.86 |
| Pigs |  | 40 min | 777.45 ±77.80 | 1297.24±163.44 | 1102.68±169.99 | 2506.76±299.50 | 2095.18±85.92 | 503.76±30.29 | 1239.14±40.58 | 109.32±63.37 | 1974.00±87.81 |
| Pigs |  | 1 h | 238.00±52.28 | 1192.35±255.30 | 764.88±509.67 | 1756.50±515.31 | 2044.50±499.78 | 251.67±20.39 | 1255.76±600.46 | 182.69±127.95 | 554.82±65.03 |
| Pigs |  | 2 h | 281.43±81.27 | 486.35±61.56 | 305.28±125.61 | 1056.45±326.39 | 752.40±255.50 | 533.30±8.78 | 1368.86±740.88 | 93.30±48.93 | 799.06±242.58 |
| Pigs |  | 4 h | 169.06±3.55 | 72.41±58.90 | 121.63±16.84 | 591.78±333.44 | 295.01±37.53 | 514.94±174.85 | 183.74±47.78 | 106.53±64.85 | 903.32±52.78 |
| Pigs | 2 | 20 min | 830.37±42.45 | 614.55±51.48 | 1426.68±73.15 | 1020.32±73.15 | 552.09±54.17 | 893.44±82.18 | 2036.12±206.45 | 348.57±47.70 | 111.26±36.45 |
| Rats | 2 | Dead time | 69.83±10.63 | 172.47±68.93 | 150.80±65.89 | 251.87±29.79 | 300.95±124.69 | 44.01±12.66 | 78.19±15.63 | 101.98±27.32 | 71.90±13.22 |

Note: The time to death of rats treated with 2 mg/kg of gelsenicine and pigs treated with 10 mg/kg of gelsenicine was approximately 15–30 min. n = 3–8, per group.

**Table S4**

Distribution of different concentrations of gelsenicine in various brain regions and spinal cords of pigs and rats.

| Animals | Dose  (mg/kg) | Tissue concentration of gelsenicine (ng/g) | | | | | | | | |
| --- | --- | --- | --- | --- | --- | --- | --- | --- | --- | --- |
|  |  | Sampling time | Hippocampus | Corpus striatum | Cortex | Brainstem | Cerebellum | Hypothalamus | Medulla oblongata | Spinal cord |
| Pigs | 10 | Dead time | 5067.95±418.5 | 6279.39±412.25 | 6254.89±347.67 | 5044.47±298.26 | 4581.26±316.87 | 4679.70±132.78 | 3734.00±205.17 | 3866.42±205.17 |
| Pigs | 6 | 20 min | 588.57±53.07 | 565.37±12.03 | 1146.25±131.05 | 690.16±193.32 | 763.80±142.95 | 806.85±153.51 | 712.10±96.53 | 734.66±87.65 |
| Pigs |  | 40 min | 1277.40±46.75 | 1305.29±63.69 | 1358.62±2.20 | 1196.30±32.43 | 1397.56±118.43 | 1307.68±102.82 | 1274.84±38.01 | 1542.42±411.13 |
| Pigs |  | 1 h | 921.32±112.05 | 657.50±132.22 | 953.34±77.05 | 776.80±188.62 | 718.17±203.04 | 776.14±137.58 | 725.22±211.88 | 735.17±233.38 |
| Pigs |  | 2 h | 329.37±80.45 | 291.75±163.21 | 369.45±94.07 | 374.79±100.14 | 345.37±81.52 | 395.87±108.96 | 393.80±130.87 | 408.76±141.10 |
| Pigs |  | 4 h | 119.01±36.23 | 114.25±1.23 | 127.86±20.99 | 214.89±114.43 | 122.33±27.83 | 152.86±25.01 | 169.06±43.13 | 274.89±100.96 |
| Pigs | 2 | 20 min | 713.29±88.08 | 761.57±74.25 | 498.29±31.38 | 510.00±2.28 | 484.90±5.60 | 1400.86±58.32 | 817.67±175.94 | 1055.62±68.24 |
| Rats | 2 | Dead time | 87.21±34.13 | 83.90±26.53 | 80.10±31.56 | 82.54±30.19 | 80.73±23.43 | 90.17±17.83 | \ | 43.48±23.87 |

Note: The time to death of rats treated with 2 mg/kg of gelsenicine and pigs treated with 10 mg/kg of gelsenicine was approximately 15–30 min. n = 3–8, per group.

**Table. S5**

Effect of 2 mg/kg gelsenicine on blood gas parameters in mice

| Test items | basic line | 2 mg/kg Gelsenicine | |
| --- | --- | --- | --- |
|  |  | 5 min | 10 min |
| PaO_2_ (mmHg) | 100.00±12.72 | 58.50±24.89 | <10±0.00 |
| PaCO_2_ (mmHg) | 25.00±2.96 | 39.00±17.06 | 113.67±15.17 |
| Hct (%) | 41.67±6.12 | 41.83±5.57 | 45.00±11.31 |
| pH | 7.30±0.02 | 7.28±0.10 | 6.84±0.02 |
| Na^+^ (mmol/L) | 153.08±3.74 | 148.50±0.70 | 167.50±17.67 |
| K^+^ (mmol/L) | 4.54±0.21 | 5.43±0.46 | 7.78±1.73 |
| Ca^2+^ (mmol/L) | 1.23±0.08 | 1.18±0.10 | 1.37±0.07 |
| Cl^-^ (mmol/L) | 127.00±4.35 | 123.50±4.92 | 128.80±8.72 |
| HCO_3_^-^(mmol/L) | 11.82±2.00 | 17.02±3.35 | 19.01±1.53 |
| AG (mmol/L) | 16.25±0.35 | 17.17±3.75 | 29.16±9.66 |

Abbreviations: Pa, partial pressure; Hct, hematocrits; AG, anion gap. n = 3 mice/group.

**Table. S6**

Effects of 6 mg/kg gelsenicine by gavage on blood gas parameters of pigs.

| Test items | basic line | 20 min | 40 min | 1 h | 2 h |
| --- | --- | --- | --- | --- | --- |
| PaO_2_ (mmHg) | 527.0 ± 82.3 | 425.4 ± 57.2 | 287.6 ± 63.5 | 309.4 ± 87.9 | 342.8 ± 45.3 |
| PaCO_2_ (mmHg) | 22.7 ± 6.6 | 38.3 ± 17.7 | 106.7 ± 8.8 | 65.9 ± 13.2 | 40.6 ± 1.3 |
| Hct (%) | 26.3 ± 4.0 | 36.0 ± 2.5 | 37.0 ± 2.8 | 32.5 ± 3.5 | 32.5 ± 0.7 |
| pH | 7.5 ± 0.1 | 7.3 ± 0.1 | 6.9 ± 0.3 | 7.2 ± 0.3 | 7.1 ± 0.1 |
| Na^+^ (mmol/L) | 139.0 ± 4.2 | 143.0 ± 3.4 | 140.5 ± 3.5 | 139.0 ± 1.4 | 142.5 ± 5.0 |
| K^+^ (mmol/L) | 6.0 ± 0.4 | 5.6 ± 0.7 | 5.3 ± 0.1 | 5.5 ± 0.6 | 5.5 ± 0.1 |
| Ca^2+^ (mmol/L) | 1.3 ± 0.1 | 1.3 ± 0.0 | 1.4 ± 0.1 | 1.4 ± 0.1 | 1.5 ± 0.0 |
| Cl^-^ (mmol/L) | 106.8±1.3 | 110.5 ± 1.0 | 105.0 ± 1.4 | 102.0 ± 2.8 | 108.5 ± 10.6 |
| HCO_3_^-^ (mmol/L) | 15.0 ± 3.0 | 15.7 ± 3.1 | 19.3 ± 6.9 | 25.5 ± 2.9 | 19.9 ± 12.0 |
| AG (mmol/L) | 22.8 ± 7.1 | 22.8±1.9 | 21.5±7.8 | 17.0±7.1 | 19.5±5.0 |

Abbreviations: Pa, partial pressure; Hct, hematocrits; AG, anion gap. n = 3 pigs.
