# Supplementary material for: Hypoxia tolerance determine differential gelsenicine-induced neurotoxicity between pig and mouse
Source: BMC Med. 2025 Mar 12;23:156. doi: 10.1186/s12916-025-03984-5 (PMC11905507; doi:10.1186/s12916-025-03984-5)
Supplement: Supplementary file 3 — Additional file 3: Table S7. Effect of glycine and NMDA on respiratory inhibition induced by gelsenicine. n=3 mice/group. [file 12916_2025_3984_MOESM3_ESM.docx]

**Table S7**

Effect of glycine and NMDA on respiratory inhibition induced by gelsenicine.

| Test items | basic line | 0.24 mg/kg Gelsenicine | | 25 mg/kg NMDA + 0.24 mg/kg Gelsenicine | | | 25 mg/kg NMDA | 1600 mg/kg glycine + 0.24 mg/kg Gelsenicine | | | 1600 mg/kg glycine |
| --- | --- | --- | --- | --- | --- | --- | --- | --- | --- | --- | --- |
|  |  | 5 min | 10 min | 5 min | 10 min | 30 min | 10min | 5 min | 10 min | 30 min | 10 min |
| PaO_2_ (mmHg) | 91.00±18.52 | 47.50±13.50 | 10.00±0.10 | 58.00±0.00 | 92.50±23.50 | 78.00±5.66 | 108.00±12.33 | 44.00±7.07 | 70.50±2.12 | 98.00±8.16 | 101.00±14.84 |
| PaCO_2_ (mmHg) | 18.45±1.48 | 58.50±3.00 | 102.93±8.16 | 52.15±2.90 | 59.10±13.10 | 34.10±1.13 | 37.05±13.93 | 27.80±3.36 | 35.20±6.92 | 21.30±4.36 | 24.70±17.80 |
| Hct (%) | 37.33±1.15 | 35.50±2.50 | 37.00±12.29 | 35.50±6.36 | 43.33±2.08 | 26.50±0.71 | 31.50±3.54 | 38.00±5.65 | 35.00±4.33 | 47.00±0.53 | 48.00±2.81 |
| pH | 7.33±0.04 | 7.17±0.01 | 6.86±0.07 | 7.20±0.04 | 7.18±0.08 | 7.30±0.06 | 7.33±0.03 | 7.27±0.05 | 7.18±0.05 | 7.28±0.04 | 7.33±0.10 |
| Na^+^ (mmol/L) | 150.30±2.52 | 148.00±0.00 | 155.00±1.73 | 153.00±0.00 | 152.33±7.02 | 150.00±0.00 | 148.50±0.71 | 155.50±0.70 | 161.00±6.06 | 156.00±0.94 | 154.00±7.16 |
| K^+^ (mmol/L) | 4.63±0.21 | 5.75±0.45 | 6.55±0.21 | 5.25±0.07 | 4.77±0.31 | 5.3±0.14 | 4.05±0.21 | 5.65±1.90 | 5.40±0.22 | 5.50±0.41 | 5.50±0.34 |
| Ca^2+^ (mmol/L) | 1.32±0.20 | 1.28±0.02 | 1.32±0.05 | 1.30±0.06 | 1.33±0.05 | 1.43±0.02 | 1.31±0.06 | 1.09±0.11 | 1.28±0.02 | 1.40±0.11 | 1.24±0.39 |
| Cl^-^ (mmol/L) | 124.00±4.24 | 119.50±2.54 | 122.67±3.51 | 119.00±0.00 | 122.33±6.50 | 120.00±2.12 | 119.00±1.41 | 123.50±0.70 | 127.00±1.78 | 135.00±3.53 | 128.00±6.94 |
| HCO_3_^-^ (mmol/L) | 13.23±1.91 | 20.85±0.55 | 17.93±2.21 | 20.50±3.04 | 21.27±4.76 | 16.25±1.48 | 18.85±5.72 | 12.70±0.00 | 13.00±4.76 | 9.70±4.38 | 12.70±4.84 |
| AG (mmol/L) | 16.50±3.54 | 13.50±2.5 | 22.33±2.52 | 19.00±2.83 | 13.33±4.51 | 18.05±0.71 | 14.00±5.66 | 24.50±2.12 | 26.00±2.33 | 17.00±0.12 | 18.00±3.41 |

Abbreviations: Pa, partial pressure; Hct, hematocrits; AG, anion gap. n=3 mice/group.
