# Supplementary figures and images for: Hypoxia tolerance determine differential gelsenicine-induced neurotoxicity between pig and mouse
Source: BMC Med. 2025 Mar 12;23:156. doi: 10.1186/s12916-025-03984-5 (PMC11905507; doi:10.1186/s12916-025-03984-5)

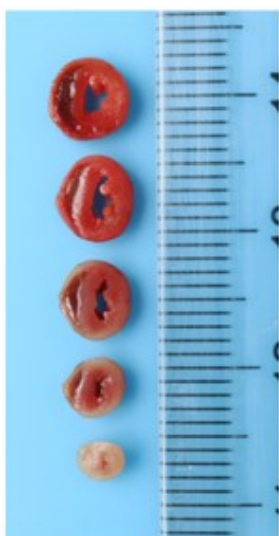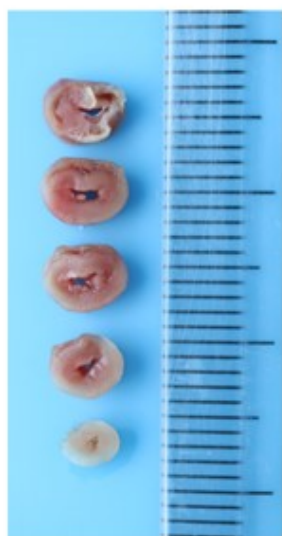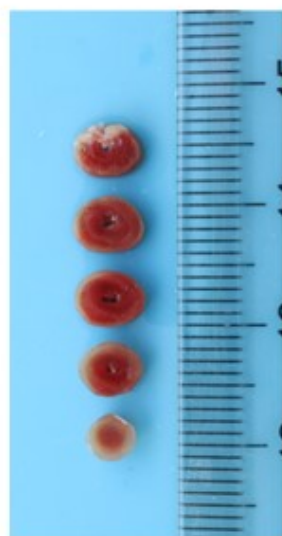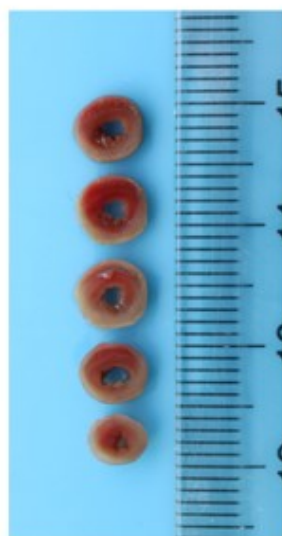

Control

Gelsenicine

NMDA + Gelsenicine

Glycine + Gelsenicine

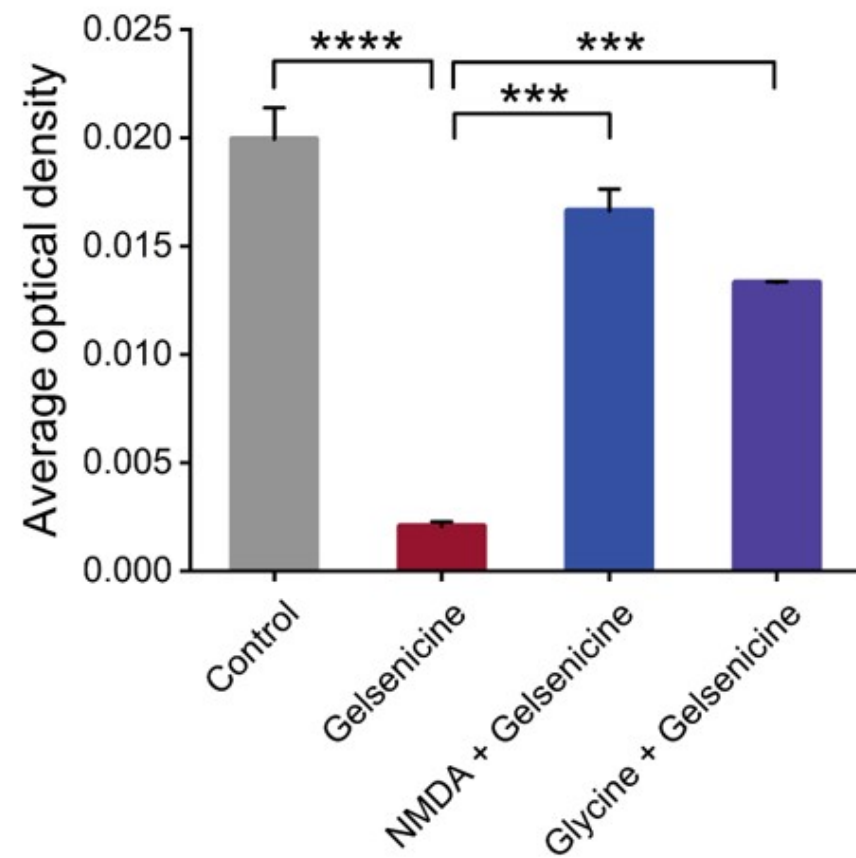

Supplement: Supplementary file 4 — Additional file 4: Figure S1. Representative heart sections for infarct size assessment by histological TTC staining and quantification of TTC-stained infarcts. Gelsenicine increased myocardial hypoxia-ischemia injury in the mice. NMDA and glycine preconditioning attenuated the damage induced by gelsenicine and improved cardiac function. Mice in gelsenicine groups received an i.p. injection of 0.24 mg/kg gelsenicine, control mice were injected with normal saline. Mice in the Glycine/NMDA + Gelsenicine groups were pre-treated with either 1600 mg/kg glycine or 25 mg/kg NMDA, respectively, prior to receiving the identical dose of 0.24 mg/kg gelsenicine. Data are represented as mean ± SD. *** P < 0.001, ****P < 0.0001 based on one-way ANOVA or unpaired t tests. n=3 mice/group. [file 12916_2025_3984_MOESM4_ESM.pdf]
