# Supplementary material for: Hypoxia tolerance determine differential gelsenicine-induced neurotoxicity between pig and mouse
Source: BMC Med. 2025 Mar 12;23:156. doi: 10.1186/s12916-025-03984-5 (PMC11905507; doi:10.1186/s12916-025-03984-5)
Supplement: Supplementary file 5 — Additional file 5: Table S8. Statistical analysis of changes in phosphorylated peptides. Mice in the 10 min and death group were intraperitoneally injected with 0.2 mg/kg gelsenicine, while the control mice were treated with equal volumes of normal saline. All the nine mice were euthanized. Abbreviations: H, hippocampus; B, brainstem. S, striatum. [file 12916_2025_3984_MOESM5_ESM.docx]

**Table S8**

Statistical analysis of changes in phosphorylated peptides.

| The comparison group | up-regulated（> 1.2） | down-regulated（< 0.833） |
| --- | --- | --- |
| 10minH/ConH | 1346 | 3 |
| DeathH/10minH | 1498 | 68 |
| DeathH/ConH | 5978 | 16 |
| 10minB/ConB | 379 | 5 |
| DeathB/10minB | 45 | 47 |
| DeathB/ConB | 267 | 53 |
| 10minS/ConS | 7 | 25 |
| DeathS/10minS | 20 | 32 |
| DeathS/ConS | 34 | 6 |

Note: Mice in the 10 min and death group were intraperitoneally injected with 0.2 mg/kg gelsenicine, while the control mice were treated with equal volumes of normal saline. All the nine mice were euthanized.

Abbreviations: H, hippocampus; B, brainstem. S, striatum.
