# Supplementary material for: Hypoxia tolerance determine differential gelsenicine-induced neurotoxicity between pig and mouse
Source: BMC Med. 2025 Mar 12;23:156. doi: 10.1186/s12916-025-03984-5 (PMC11905507; doi:10.1186/s12916-025-03984-5)

Fig5 A

GABA$_A$Rβ1 (1:1000)

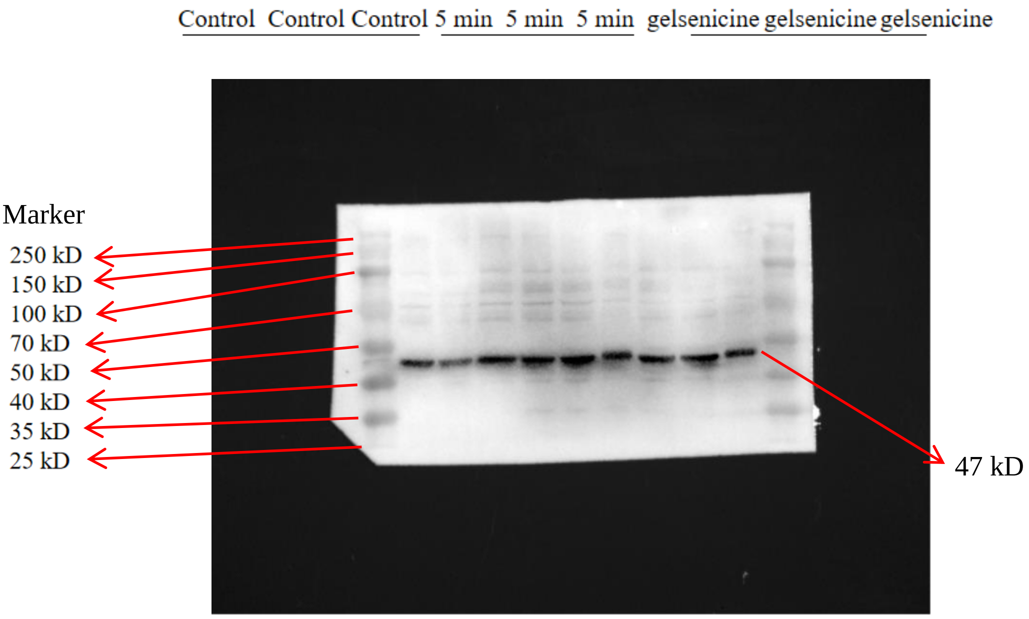

β-actin (1:80000)

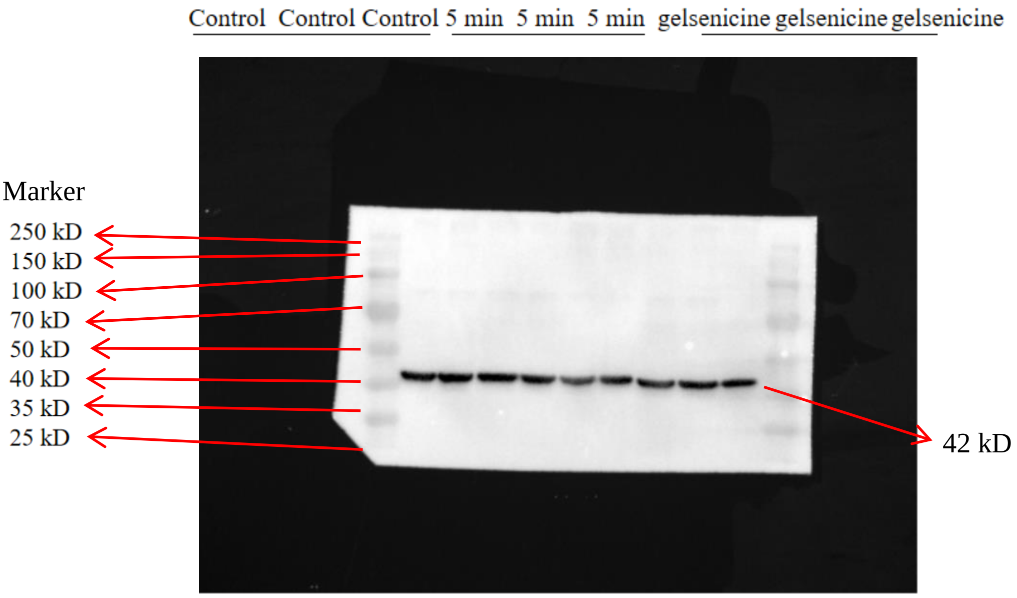

Fig5 G

GluN2A (1:1000)

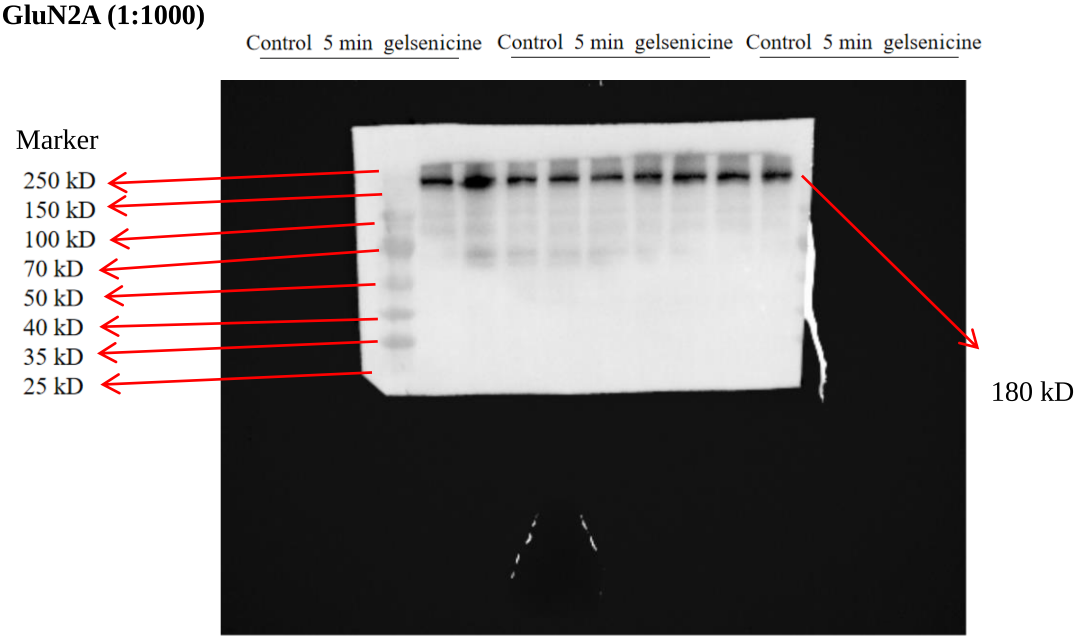

β-actin (1:80000)

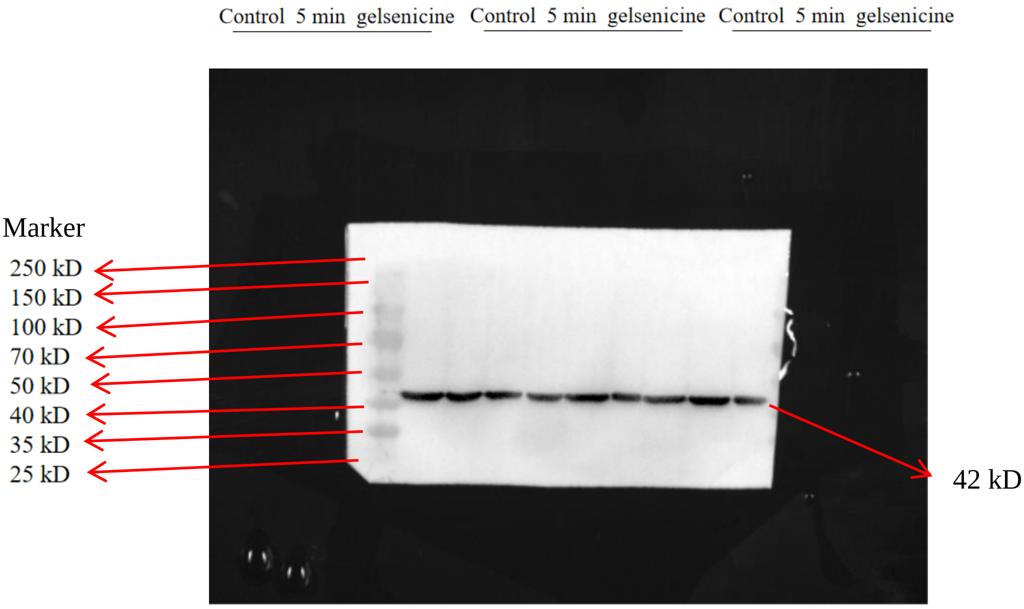

Fig5 H

GluN2B (1:1000)

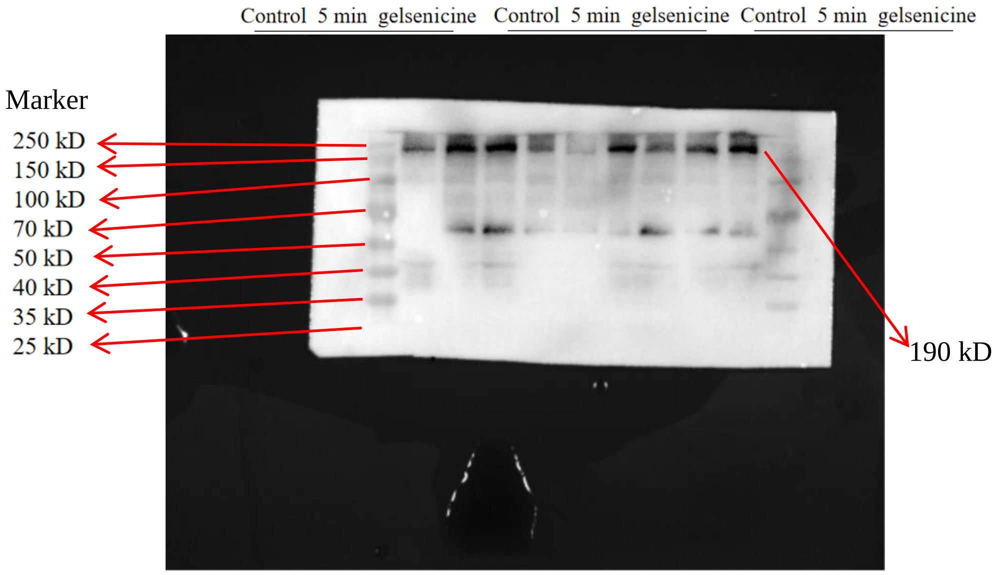

β-actin (1:80000)

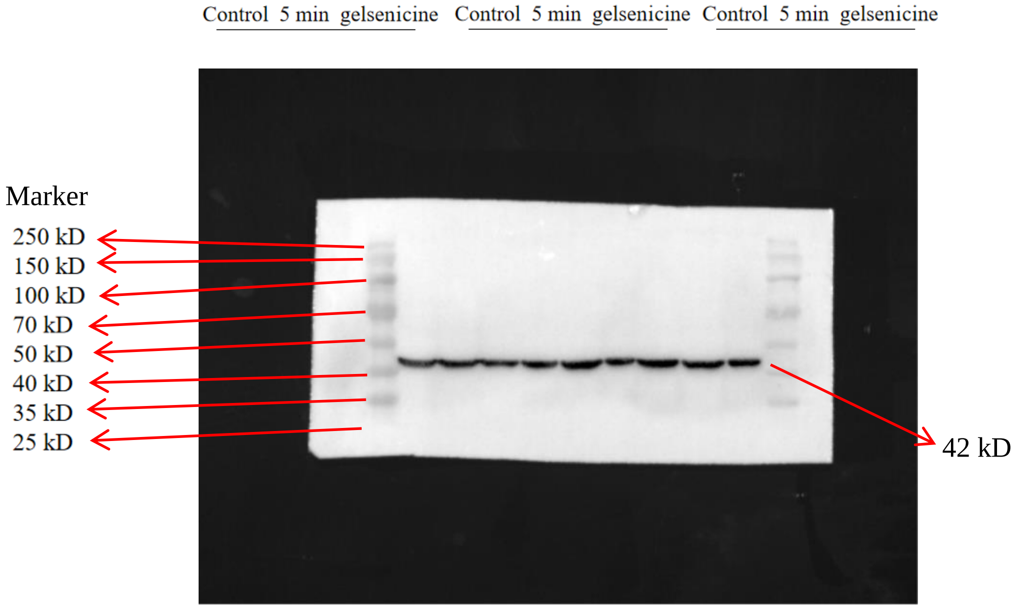

Fig6 G

GluN2A (1:1000)

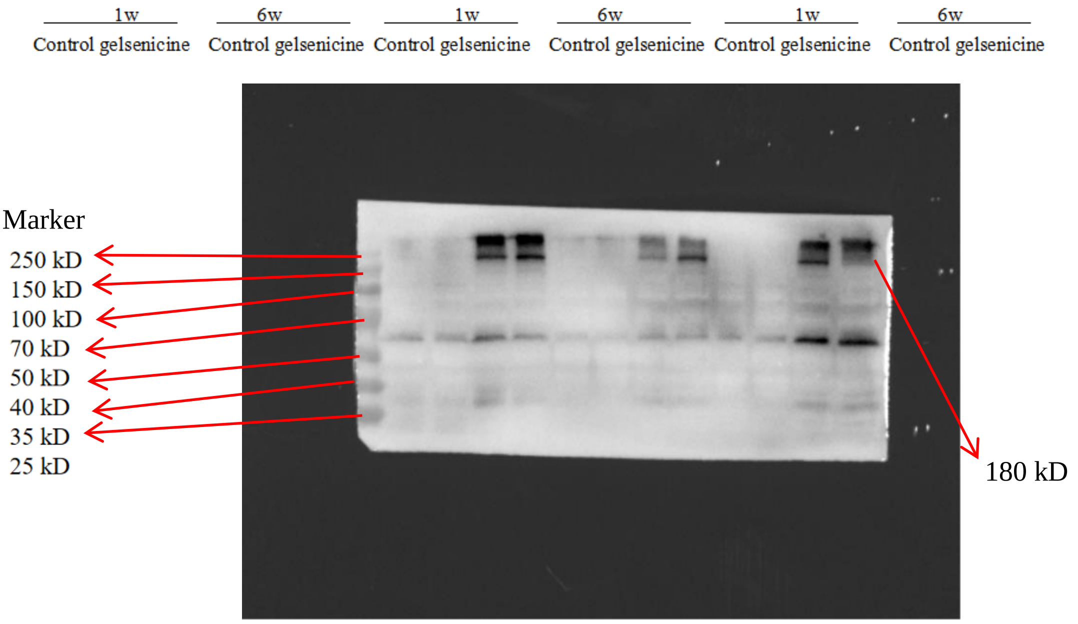

β-actin (1:80000)

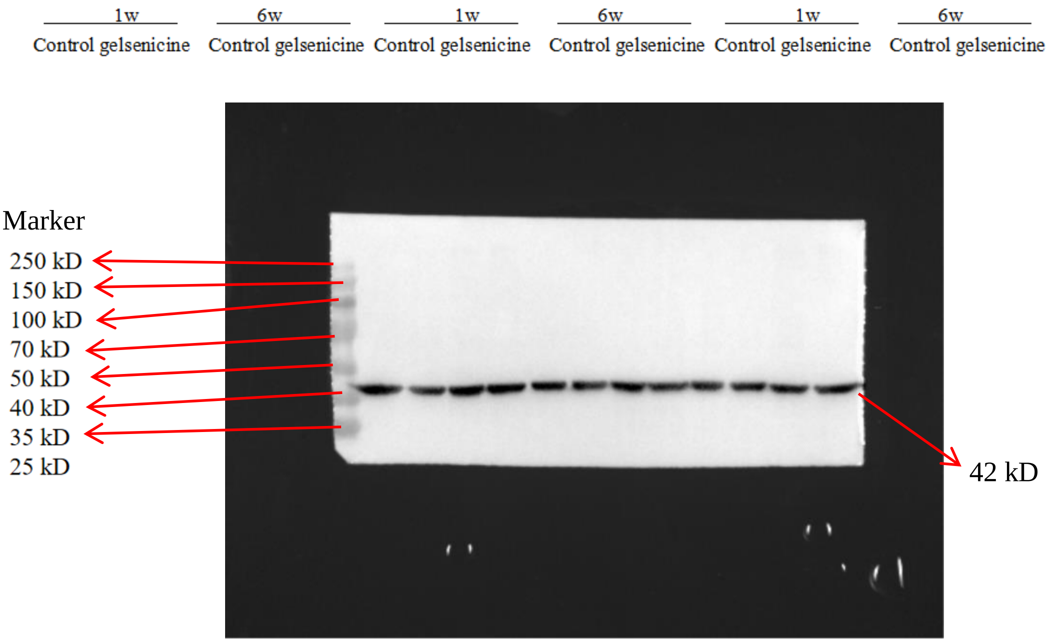

Fig6 H

GluN2B (1:1000)

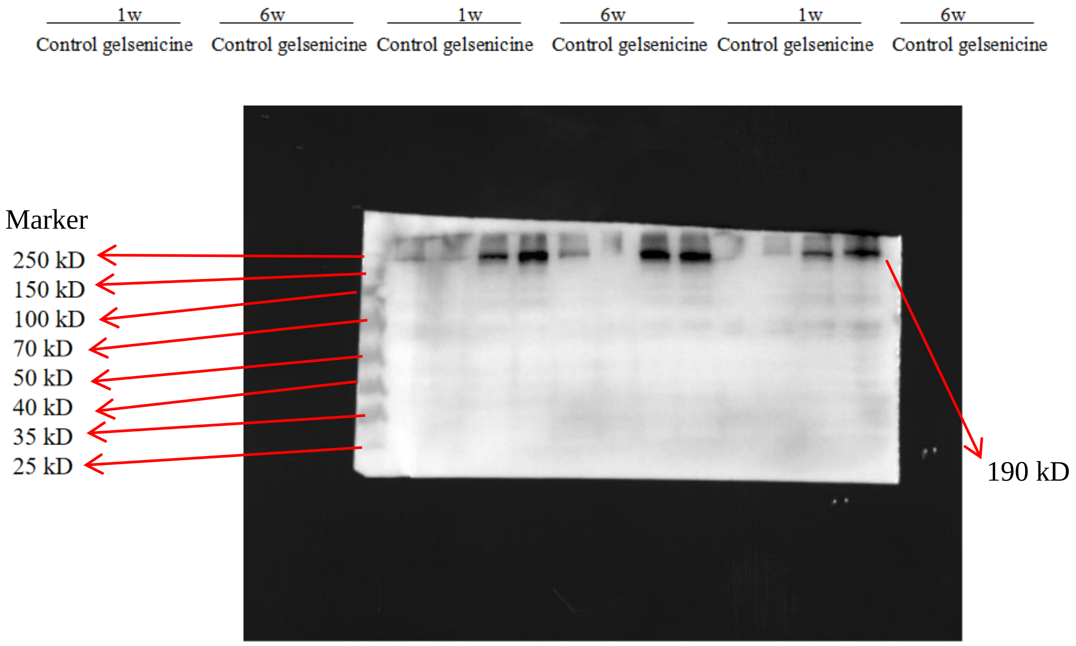

β-actin (1:80000)

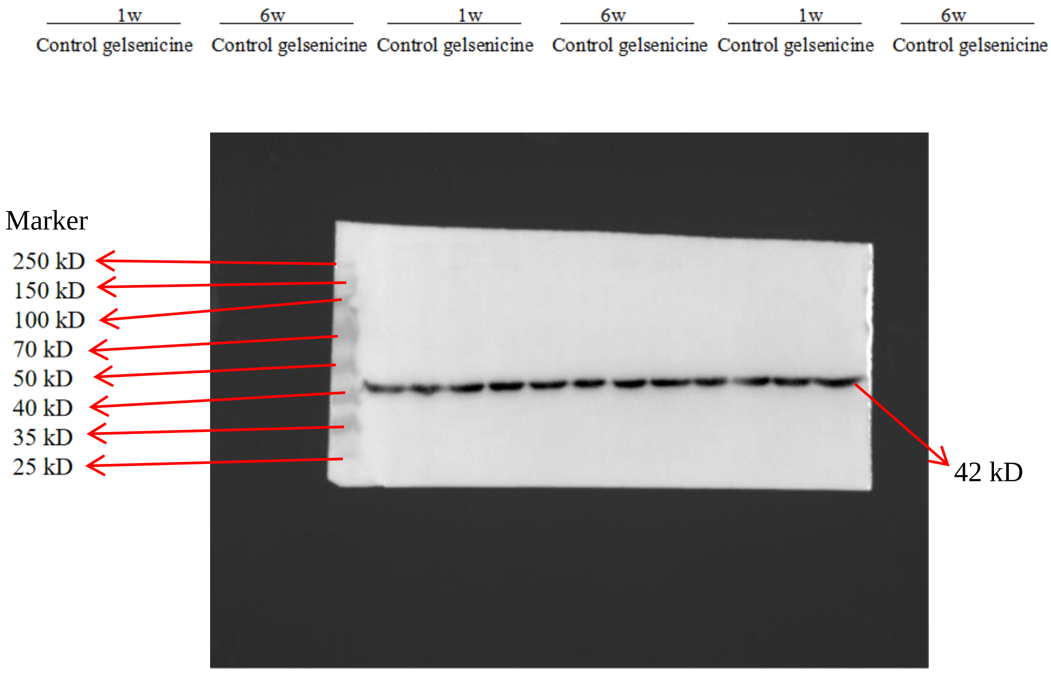

Fig6 I

GluN2A (1:1000)

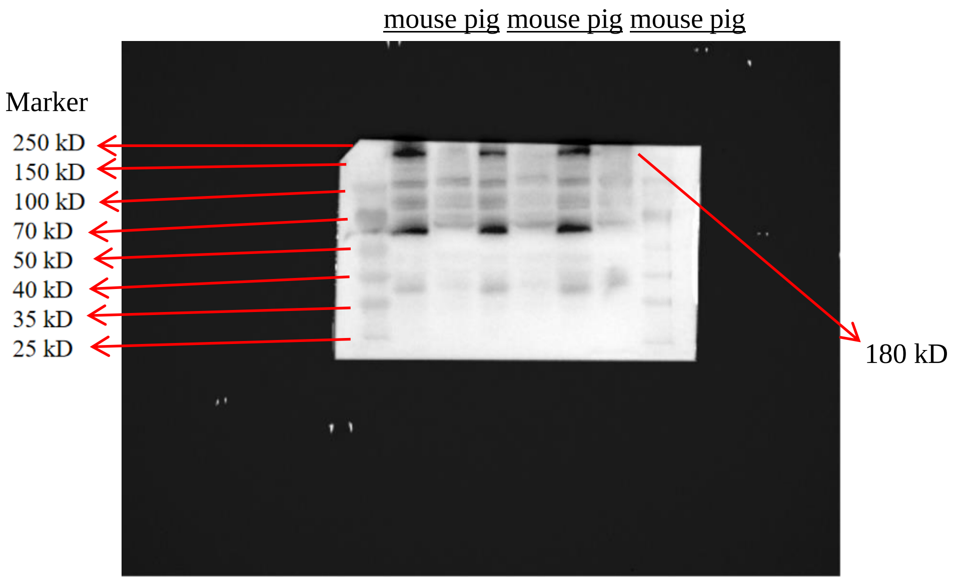

β-actin (1:80000)

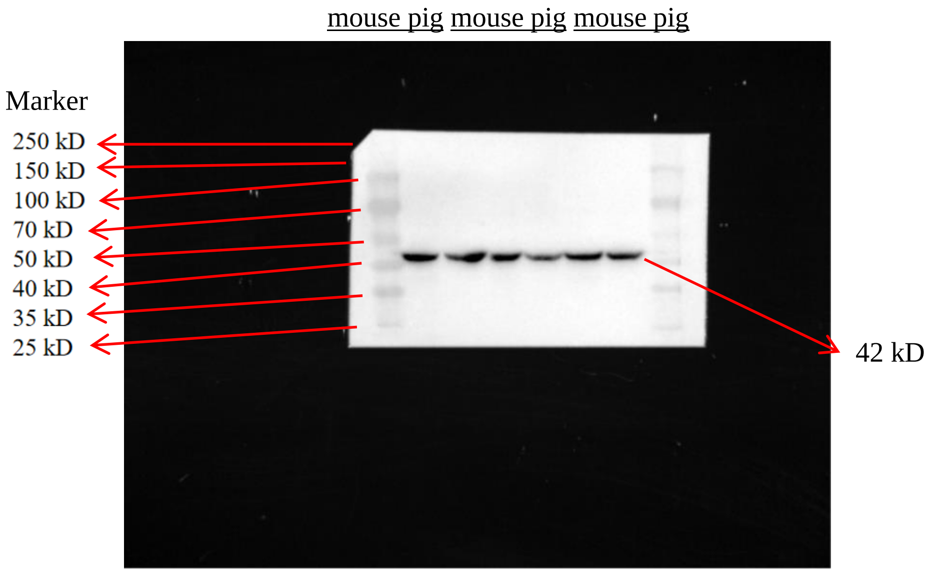

Fig6 J

GluN2B (1:1000)

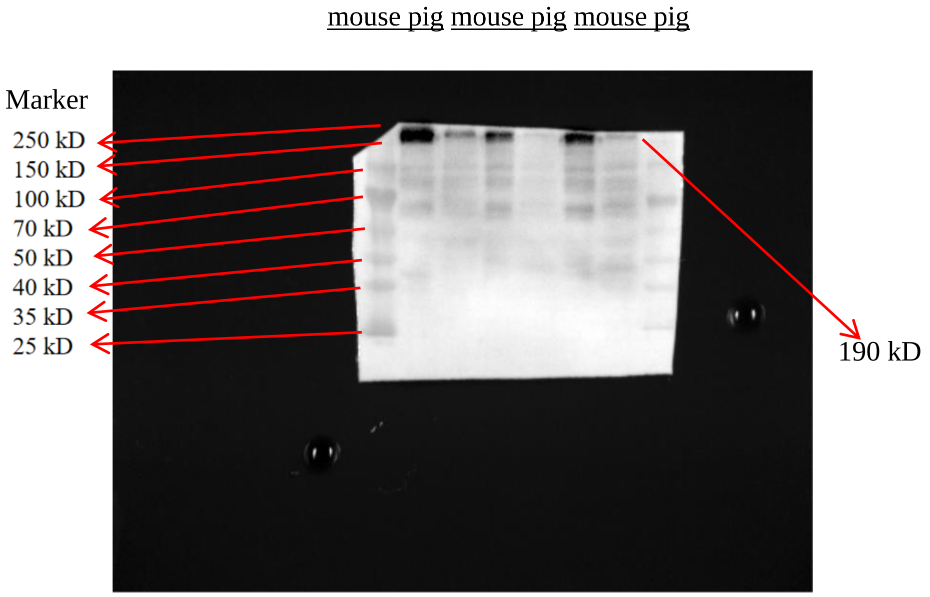

β-actin (1:80000)

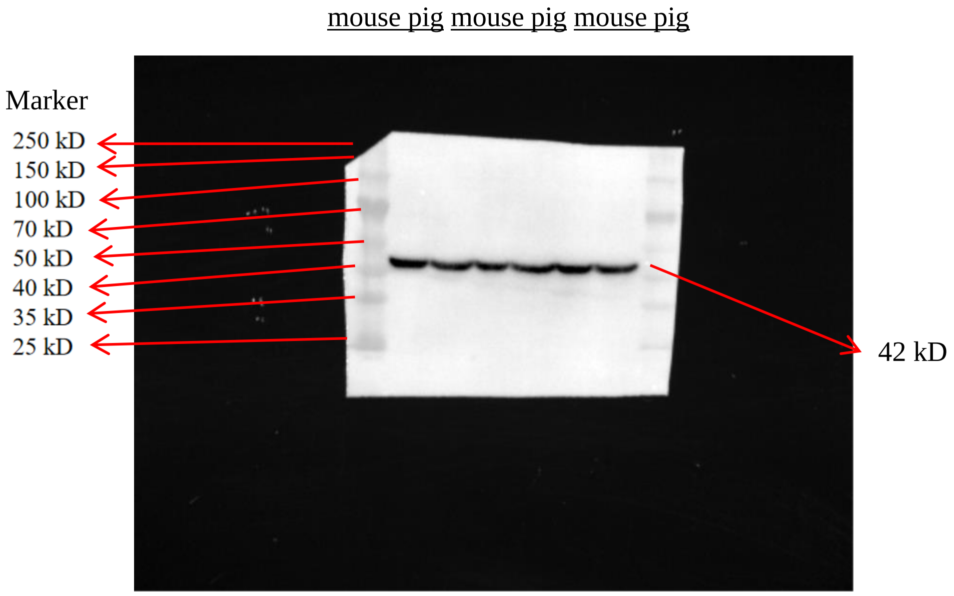

Supplement: Supplementary file 6 — Additional file 6. The original, uncropped gels. [file 12916_2025_3984_MOESM6_ESM.pdf]
