# Supplementary material for: Hypoxia tolerance determine differential gelsenicine-induced neurotoxicity between pig and mouse
Source: BMC Med. 2025 Mar 12;23:156. doi: 10.1186/s12916-025-03984-5 (PMC11905507; doi:10.1186/s12916-025-03984-5)
Supplement: Supplementary file 7 — Additional file 7. The Arrive checklist. [file 12916_2025_3984_MOESM7_ESM.docx]

**Major Resources Table**

In order to allow validation and replication of experiments, all essential research materials listed in the Methods should be included in the Major Resources Table below. Authors are encouraged to use public repositories for protocols, data, code, and other materials and provide persistent identifiers and/or links to repositories when available. Authors may add or delete rows as needed.

**Animals (in vivo studies)**

| **Species** | **Vendor or Source** | **Background Strain** | **Sex** | **Persistent ID / URL** |
| --- | --- | --- | --- | --- |
| Mice (neonatal and adult) | Slake Jingda Laboratory Animal Technology Company (Hunan Province) | ICR mice | Male | http://www.hnsja.com/products_detail/c-_detailId%3D1006227185370648576.html |
| Rats | Slake Jingda Laboratory Animal Technology Company (Hunan Province) | SD rats | Male | http://www.hnsja.com/products_detail/c-_detailId%3D999280774306418688.html |
| Pigs | Xin Guangan Xiangda Co., Ltd. | Ternary hybrid piglets | Male | N/A |

**Genetically Modified Animals**

|  | **Species** | **Vendor or Source** | **Background Strain** | **Other Information** | **Persistent ID / URL** |
| --- | --- | --- | --- | --- | --- |
| **Parent - Male** | None |  |  |  |  |
| **Parent - Female** | None |  |  |  |  |

**Antibodies**

| **Target antigen** | **Vendor or Source** | **Catalog #** | **Working concentration** | **Lot # (preferred but not required)** | **Persistent ID / URL** |
| --- | --- | --- | --- | --- | --- |
| NMDA Receptor 2A (GluN2A) Antibody | Cell Signaling | Cat#4205 | 1:1000 |  | https://www.cellsignal.cn/products/primary-antibodies/nmda-receptor-2a-glun2a-antibody/4205 |
| NMDA Receptor 2B (GluN2B) Antibody | Cell Signaling | Cat#4207 | 1:1000 |  | https://www.cellsignal.cn/products/primary-antibodies/nmda-receptor-2b-glun2b-antibody/4207 |
| GABA A Receptor beta 2 (GABRB2) Rabbit pAb | ABclonal | Cat#A1876 | 1:1000 |  | https://abclonal.com.cn/catalog/A1876 |
| β-Actin Rabbit mAb | ABclonal | Cat#AC026 | 1:80000 |  | https://abclonal.com.cn/catalog/AC026 |
| HRP conjugated goat anti rabbit IgG (H + L) | Biodragon | BF03008 | 1:5000 |  | https://www.biodragon.cn |
| Rabbit anti-c-Fos | Abcam | #ab222699 | 1:1000 |  | https://www.abcam.cn/products/primary-antibodies/c-fos-antibody-epr21930-238-ab222699.html |
| Goat Anti-Rabbit IgG H&L (HRP) | Abcam | #ab205718 | 1:2000 |  | https://www.abcam.cn/products/secondary-antibodies/goat-rabbit-igg-hl-hrp-ab205718.html |

**DNA/cDNA Clones**

| **Clone Name** | **Sequence** | **Source / Repository** | **Persistent ID / URL** |
| --- | --- | --- | --- |
| None |  |  |  |
|  |  |  |  |
|  |  |  |  |

**Cultured Cells**

| **Name** | **Vendor or Source** | **Sex (F, M, or unknown)** | **Persistent ID / URL** |
| --- | --- | --- | --- |
| HEK-293 cell lines stably expressing GluN1/GluN2A or GluN1/GluN2B receptors | ICE Bioscience Inc. | unknown | https://en.ice-biosci.com/ |
|  |  |  |  |
|  |  |  |  |

**Data & Code Availability**

| **Description** | **Source / Repository** | **Persistent ID / URL** |
| --- | --- | --- |
| None |  |  |
|  |  |  |
|  |  |  |

**Other**

| **Description** | **Source / Repository** | **Persistent ID / URL** |
| --- | --- | --- |
| None |  |  |
|  |  |  |
|  |  |  |

**ARRIVE GUIDELINES**

The ARRIVE guidelines (<https://arriveguidelines.org/>) are a checklist of recommendations to improve the reporting of research involving animals. Key elements of the study design should be included below to better enable readers to scrutinize the research adequately, evaluate its methodological rigor, and reproduce the methods or findings.

**Study Design**

| **Groups** | **Sex** | **Age** | **Number (prior to experiment)** | **Number (after termination)** | **Littermates**  **(Yes/No)** | **Other description** |
| --- | --- | --- | --- | --- | --- | --- |
| Control group | Male | 6-8-week-old (adult) | 14 | 14 | Yes | Control mice were injected with saline. |
| 0.24 mg/kg Gelsenicine group | Male | 6-8-week-old (adult) | 14 | 0 | Yes | Mice received an i.p. injection of 0.24 mg/kg gelsenicine. |
| 1600 mg/kg Glycine + 0.24 mg/kg Gelsenicine group | Male | 6-8-week-old (adult) | 14 | 10 | Yes | Mice in the Glycine + Gelsenicine groups were pre-treated with 1600 mg/kg glycine, respectively, prior to receiving the identical dose of 0.24 mg/kg gelsenicine. |
| 25 mg/kg NMDA + 0.24 mg/kg Gelsenicine group | Male | 6-8-week-old (adult) | 14 | 10 | Yes | Mice in the NMDA + Gelsenicine groups were pre-treated with 25 mg/kg NMDA, respectively, prior to receiving the identical dose of 0.24 mg/kg gelsenicine. |

**Sample Size:** Please explain how the sample size was decided Please provide details of any a *prior* sample size calculation, if done.

None

**Inclusion Criteria**

None

**Exclusion Criteria**

None

**Randomization**

None

**Blinding**

None
